# The role of SLC2A1 in lung adenocarcinoma: From tumorigenesis to patient survival

**DOI:** 10.1371/journal.pone.0324043

**Published:** 2025-08-18

**Authors:** Zijun Xiao, Qinqin Long, Jiaxing Liao, Fengdie Huang, Lusheng Liao, Mingyou Dong

**Affiliations:** 1 Modern Industrial College of Biomedicine and Great Health, Youjiang Medical University for Nationalities, Baise, China; 2 Department of Pathology, the Affiliated Hospital of Youjiang Medical University for Nationalities, Key Laboratory of Molecular Pathology in Tumor of Guangxi Higher Education Institutes, Baise, China; 3 Clinical Laboratory of Hechi Traditional Chinese Medicine Hospital, Hechi, China; 4 Clinical Laboratory, The People’s Hospital of Baise, Baise, China; BMSCE: BMS College of Engineering, INDIA

## Abstract

**Objective:**

Our study aimed at systematically exploring the effect of the solute carrier family 2 Member (*SLC2A*) genes family on the prognosis and immune landscape of lung adenocarcinoma (LUAD) patients. Furthermore, we sought to determine the *SLC2A1* function in LUAD initiation and progression through *in vivo* and *in vitro* experiments.

**Methods:**

A comprehensive bioinformatics analysis was conducted utilizing online tools and software, including R packages, Gene Set Cancer Analysis (GSCA), cBio Cancer Genomics Portal (cBioPortal), GeneMANIA, STRING, and Xiantao Academic Online databases, to assess the functional implications of the *SLC2A* gene family in LUAD. Concurrently, *in vivo* and *in vitro* experiments at the cellular and animal levels were conducted to ascertain the effects of *SLC2A1* gene knockout on LUAD development.

**Results:**

Compared to normal tissues, the *SLC2A* gene family exhibited significant upregulation across various tumor types, including LUAD, with a low mutation frequency in LUAD. *SLC2A1* and *SLC2A7* emerged as prognostic biomarkers for LUAD. The receiver operating characteristic (ROC) curve analysis revealed high diagnostic accuracy of *SLC2A1* for LUAD. A significant negative correlation was observed between *SLC2A1* expression and DNA methylation levels in LUAD, and the gene was closely linked to cellular processes such as cell nuclear division, DNA replication, and metabolism. Moreover, *SLC2A1* expression was strongly linked to immune infiltration and regulation across different tumor types. *In vitro* and *in vivo* experiments showcased that *SLC2A1* inhibition significantly hampered LUAD A549 cell proliferation, migration, and invasion capabilities, as well as tumor growth in nude mice. Finally, our study demonstrated that reduced *SLC2A1* expression influenced the expression of molecules within the P53 signaling pathway.

**Conclusions:**

This study elucidates the functional role of the *SLC2A* gene family in the pathogenesis of LUAD, underscoring the importance of *SLC2A1* in LUAD diagnosis, prognosis, and immune response, and presenting *SLC2A1* as a promising biomarker for LUAD.

## Introduction

Globally, lung cancer is the deadliest malignancy, claiming approximately 1.8 million lives annually [1]. In 2022, it accounted for roughly 2.5 million new cases, ranking as the primary cause of cancer morbidity and mortality among 36 tumor types, presenting a substantial threat to human health [2]. Lung adenocarcinoma (LUAD) has emerged as the predominant histological lung cancer subtype, surpassing lung squamous cell carcinoma in incidence [3]. Originating primarily from bronchial mucosal epithelial cells and occasionally from the mucous glands of the main bronchi, LUAD’s complex pathogenesis has hindered efforts to reduce its high mortality rate [4]. The lack of reliable biomarkers forlung adenocarcinoma further limits the accuracy of prognostic prediction [5]. Consequently, the identification of novel tumor markers is imperative for advancing the precision treatment of LUAD and improving patient outcomes.

Tumor cells display distinct metabolic features compared to their normal counterparts, relying heavily on glycolysis and glucose uptake to generate sufficient ATP for their energetic demands. This metabolic shift is facilitated by the solute carrier family 2 (SLC2A), comprising 14 genes encoding glucose transporter proteins (GLUTs) [6]. Notably, GLUTs are integral members belonging to the broader facilitator superfamily of membrane transport proteins [7]. Encoded SLC2A proteins can be categorized into three groups based on their primary function: GLUT1–4/14 primarily mediate hexose transport across cellular membranes, while GLUT5/7/9/11, as well as GLUT6/8/10/12/13, predominantly promote glucose uptake [8]. Previous studies have linked the expression of SLC2A members to various malignant tumors. Notably, SLC2A1, also known as GLUT1, is a critical component of cellular energy metabolism and has been involved in development and progression of multiple cancer [9–11]. Bioinformatics analyses have identified SLC2A1 as a potential biomarker for LUAD [12]. However, the precise impact of the *SLC2A* gene family on LUAD prognosis and survival remains to be fully elucidated.

In the present study, a comprehensive multi-omics analysis was conducted to ascertain the prognostic and biological significance of the *SLC2A* gene family in LUAD. In addition, we evaluated the differential expression of the *SLC2A* gene family between normal and LUAD tissues. Subsequently, the prognostic and diagnostic utility of *SLC2A* gene expressions in LUAD was determined. The relationship between *SLC2A1* expression as well as tumor immune infiltration and immune checkpoint molecules, was also determined. To elucidate the functional role of *SLC2A1*, CRISPR-Cas9-mediated knockout of *SLC2A1* in LUAD cell lines was performed, followed by *in vitro* assays including Western blotting, RT-qPCR, colony formation, CCK-8, scratch, and Transwell invasion assays to evaluate cell proliferation, migration, and invasion. *SLC2A1* tumorigenicity was assessed *in vivo* through nude mouse xenograft models. Furthermore, The Cancer Genome Atlas (TCGA) database was deployed to uncover key signaling pathways potentially regulated by *SLC2A1* in LUAD pathogenesis. Collectively, our results present novel perspectives into the molecular mechanisms underlying *SLC2A1*’s role in LUAD, with implications for early diagnosis, prognostication, and immunotherapy development.

## Materials and Methods

### Data acquisition and analysis

The Xiantao Academic Online platform (https://www.xiantaozi.com/) was employed to examine RNA sequencing (RNA-seq) data from 598 LUAD patients in TCGA. This dataset comprised 539 LUAD tumor samples and 59 normal lung tissue samples. We also collected clinical data for these LUAD patients, including various prognostic indicators, particularly Tumor Node Metastasis (TNM) stage, pathological stage, residual tumor status, age, gender, and overall survival (OS). Furthermore, differential expression of *SLC2A1* was assessed across five distinct datasets: GSE19804 [13], GSE6857 [14], GSE10072 [15], GSE116959 [16], and GSE140797 [17] retrieved from Xiantao Academic.

### Investigation of the differential expression of *SLC2A*s in LUAD

A multifaceted approach was employed to investigate the expression patterns of the *SLC2A* gene family (*SLC2A1-14*) across various human tissues. First, we deployed the “GTEx Expression” module within the Gene Set Cancer Analysis (GSCA) database (http://bioinfo.life.hust.edu.cn/web/GSCALite/) [18] to assess each family member’s expression in normal human tissues. Subsequently, the “General” module of the Gene Expression Profiling Interactive Analysis (GEPIA) database (http://gepia.cancer-pku.cn/detail.php) [19] was employed to compare mRNA expression differences of SLC2A1-14 between normal tissues and 31 different tumor types. Furthermore, the Xiantao Academic Online Platform was utilized to evaluate the *SLC2A1-14* expression levels in normal tissues along with 33 types of tumor tissues using the “Group Comparison” module. This platform was further leveraged to visualize non-paired and paired expression differences of *SLC2A*s in the TCGA LUAD dataset through the “Disease vs. Non-disease” and “Paired Samples” modules.

### Analysis of genetic variants of *SLC2A*s and construction of an interaction network

The cBioPortal database (https://www.cbioportal.org/) [20] was employed to identify mutations within the *SLC2A1-14* genes in 586 LUAD samples obtained from the TCGA Firehose Legacy project. Moreover, the GeneMANIA database (https://genemania.org/) [21] was utilized to construct an interaction network for *SLC2A* genes and investigate their potential functional roles. Furthermore, we generated a protein-protein interaction (PPI) network for *SLC2A* genes utilizing the STRING database (version 12.0) (https://cn.string-db.org/) [22], with a minimum required confidence score of 0.4 and all other parameters set to default. Finally, we assessed the co-expression of *SLC2A* family members within LUAD samples using Spearman’s correlation coefficient within the “Interaction Network” module available on the Xiantao Academic Online Tools “Cloud” platform. Finally, the findings were visualized as a heatmap.

### Prognostic and diagnostic analysis of *SLC2A*s

Both univariate and multivariate Cox regression analyses were conducted through the “Clinical Significance” module of the Xiantao Academic Online Platform’s “cloud COX regression” tool to determine potential prognostic biomarkers of the SLC2A family member’s impact on LUAD. The platform’s “cloud survival curve (KM plot)” module was then employed to determine the influence of the SLC2A family on LUAD patient survival. A nomogram was constructed using the “cloud prognostic nomogram” module to predict 1-, 3-, and 5-year OS for LUAD patients. Using calibration curves generated via the “cloud calibration curve” module, the nomogram predictive accuracy was assessed. Finally, the diagnostic utility of SLC2A family members for LUAD was determined through ROC curves besides calculating the area under the curve (AUC) values with the “cloud diagnostic ROC” module.

### DNA methylation analysis

The “Methylation & expression” module of the GSCA database (https://guolab.wchscu.cn/GSCA/#/) was utilized to perform Spearman correlation analysis. This analysis explored the correlation between *SLC2A1* mRNA expression and its methylation levels in LUAD patients. Additionally, we employed the UALCAN database (https://ualcan.path.uab.edu/) [23] to assess *SLC2A1* methylation status in both normal and LUAD tissues. Furthermore, we stratified LUAD patients into diverse clinicopathological subgroups, including grade, N stage, and gender, and examined the methylation status of *SLC2A1* within each subgroup. Methylation levels were quantified via β values ranging from 0–1, with 0 representing an unmethylated state and 1 signifying complete methylation. For this analysis, high methylation was recognized as β values between 0.5 and 0.7, and low methylation was identified as β values between 0.25 and 0.3.

### Functional enrichment analysis

Using the median *SLC2A1* expression level as a cutoff, LUAD patients within the TCGA-LUAD dataset, were stratified into high and low *SLC2A1* expression groups. Differentially expressed genes (DEGs) between these cohorts were recognized via the limma R package, with an absolute log2 fold change (log2FC) threshold ≥ 1.5 and adj-*P* < 0.05. To uncover potential functional annotations of the identified *SLC2A1-*related DEGs, gene set enrichment analyses were carried out utilizing the Home for researchers website (https://www.home-for-researchers.com/#/), employing the Kyoto Encyclopedia of Genes and Genomes (KEGG) and Gene Ontology (GO) databases.

### Immune cell infiltration analysis

The CIBETSORT and ESTIMATE R packages for immune-related analysis were employed to ascertain the correlation between *SLC2A1* expressions and immune cell infiltration. The ESTIMATE Score, which combines the Immune and Stromal Scores, was deployed to quantify the immune alongside stromal components within tumors. Heatmaps were generated using the pheatmap and ggplot2 R package to visualize the relation between *SLC2A1* mRNA expression and various immune cell subpopulations. Furthermore, we utilized XianTao Academic’s interactive network module to assess the *SLC2A1* co-expression with individual immune checkpoints. This analysis employed “(cloud) correlation heatmap” and “(cloud) expression correlation scatter plot” visualizations. The association between *SLC2A1* expression and 33 types of tumor immune inhibitors and stimulants, chemokines, and their corresponding receptors was explored via data obtained from the Tumor and Immune System Interaction Database (TISIDB) (http://cis.hku.hk/TISIDB/index.php) [24].

### Cell lines

The normal human bronchial epithelial (BEAS-2B) and human LUAD (A549) cell lines were obtained from the Institute of Cell Biology, Chinese Academy of Sciences (Shanghai, China). LUAD cell lines with *SLC2A1* gene knockout were produced via CRISPR-Cas9 gene editing technology and designated as sg-SLC2A1, while the control cell line was designated as sg-NC (A549). The knockout sgRNA sequences were: sgRNA1-SLC2A1: CACCGATGATGAAGCGGCCCAGG, sgRNA2-SLC2A1: GACGATGCCCAGCTGGTGCAGGG.

### Cell line culture conditions

The cells were retrieved from the ultra-low temperature freezer and transferred to a 37°C-water bath for thawing. Subsequently, they were placed in 15 mL centrifuge tubes, mixed with 2 mL of complete culture medium, and centrifuged at 1000 rpm for 3 min employing a low-speed centrifuge. The supernatant was eliminated, and the cells were resuspended in 5 mL of complete culture medium before being transferred to T25 flasks. The flasks were incubated at 37°C in 5% CO_2_, and cell growth was monitored. Upon reaching approximately 80% confluence, the cells were passed or exposed to the next experiments.

### Reverse transcription-quantitative polymerase chain reaction (RT-qPCR)

RNA was extracted utilizing the TRIzol reagent (Invitrogen, USA), followed by quantification of its concentration and purity. Reverse transcription was then carried out per the protocol provided by TOLOBIO. The resulting cDNA was employed as a template for RT-qPCR, with human GAPDH employed as an internal reference. The qPCR reaction system was prepared in accordance with TOLOBIO’s guidelines, and amplification was carried out using a PCR instrument (HEMR, China). SLC2A1 mRNA expression was evaluated via the comparative Ct (2^-ΔΔCt^) method. The sequences of PCR primers employed are as follows: SLC2A1-F: TCATTGTGGGCATGTGCTTC, SLC2A1-R: GCTCCTCGGGTGTCTTGTCA, P53-F: ATGAGCCGCCTGAGGTTGG, P53-R: CAGTGTGATGATGGTGAGGATGG, P21-F: ACCACTGGAGGGTGACTTCGC, P21-R: CGAGGCACAAGGGTACAAGACAG, BAX-F: CAGGATGCGTCCACCAAGAAGC, BAX-R: CCCCAGTTGAAGTTGCCGTCAG, GAPDH-F: CGGGAAACTGTGGCGTGAT, GAPDH-R: TGCTCAGTGTAGCCCAGGATG.

### Western blot assay

Proteins were extracted from samples via RIPA lysis buffer and measured utilizing the BCA protein assay kit (Beyotime, China). Afterward, protein samples were exposed to electrophoresis, followed by transfer onto a methanol-activated polyvinylidene fluoride membrane incubated with the primary antibody at 4°C overnight. Subsequently, the membrane was incubated with the secondary antibody at 37°C for 30 min. Protein bands were visualized through image development, with human GAPDH serving as an internal loading control.

### Colony formation assay

Upon reaching 80% or more of their maximum length, cells were dissociated using trypsin, and counted. Subsequently, the cells were plated into each well of six-well plates with 700 cells/well. Each well was supplemented with 2 mL of Dulbecco’s Modified Eagle Medium (DMEM) culture medium composed of 10% fetal bovine serum, and the cells were incubated at 37°C in 5% CO_2_ for 13 days. After a 15- minute fixation, the cells were stained with 0.1% crystal violet for 15 minutes, photographed, and analyzed statistically.

### CCK8 assay

A cell suspension was prepared with 3,000 cells/well and incubated in a 96-well plate. Typically, 10 µL of CCK-8 reagent (DOJINDO) were introduced at 0, 24, 48, 72, and 96 h. Following 2 h incubation, the optical density (OD) was determined at 450 nm via a multifunctional microplate reader.

### EdU (5-ethynyl-2’-deoxyuridine) staining assay

Cells were cultured in 96-well plates at 4 × 10^5^ cells/well and incubated for one day. On the following day, 10 μL of EDU staining reagent was introduced to each well and incubated for 2 h. The reaction solution was prepared according to the BeyoClick™ EdU-488 Cell Proliferation Assay Kit protocol, and 50 μL was introduced to each well. Following a 30-min incubation, the cell nucleus was stained with Hoechst 33342 (Beyotime, China) per the reagent protocols and then imaged and photographed employing an inverted fluorescence microscope (Olympus, Japan).

### Wound healing assay

Cells were seeded into six-well plates at a density of 1 × 10^5^ cells/well, using DMEM supplemented with 10% fetal bovine serum. On the next day, lines were drawn on the six-well plate using a 200 µL pipette tip, followed by three washes with PBS. Next, each well was supplemented with serum-free DMEM medium and incubated for cultivation purposes. Photographs were captured at 48 h.

### Transwell migration and invasion assay

A 24-well Transwell device (Corning Costar, USA) was employed. A total of 5,000 cells/mL and 100 µL of the resulting suspension were introduced to the Transwell device upper chamber. Simultaneously, 600 µL of DMEM culture medium containing 10% fetal bovine serum was introduced to the lower chamber. Subsequently, the system was incubated for 48 h. Non-migrated cells within the upper chamber were eliminated with a cotton swab, and migrated cells in the lower chamber were fixed with 4% paraformaldehyde. The fixed cells were stained with 0.1% crystal violet to assess disparities in cell migration capacity between sg-NC and sg-SLC2A1 cells. To explore the *SLC2A1* gene knockout impact on the A549 cell’s invasive ability, an additional step was included in the experiment: Matrigel gel (Corning Costar, USA) was coated on the Transwell chamber bottom and allowed to polymerize for 2 h at room temperature. The protocol used for cell migration was replicated for the remaining steps. Cells were then imaged and counted via inverted microscopy, facilitating quantitative analysis of the invasive and migratory potential of *SLC2A1* knockout A549 cells.

### Nude mouse tumorigenicity assay

Five-week-old male mice were acquired from Guangdong Vital River Laboratory Animal Technology Co., Ltd. *SLC2A1* gene-knockout cells and the wild-type control cells were injected into the right axillary region of nude mice, respectively. The animals were acclimated in a sterile environment maintained at 22–25°C, with 40%–60% humidity and a 12-h light-dark cycle. Tumor size was quantified weekly employing a caliper, and tumor volume was calculated using the formula V = X × Y^2^/2 (where V represents tumor volume, X denotes the longest dimension, and Y is the shortest dimension). At six weeks, the mice were euthanized via cervical dislocation for tumor block removal, volume and weight measurement, and subsequent qualitative and quantitative analyses. To further elucidate the role of SLC2A1 in lung adenocarcinoma progression, we subsequently conducted hematoxylin and eosin (H&E) staining, and immunohistochemistry. The experimental protocol was approved by the Ethics Committee of Youjiang Medical University for Nationalities’s Animal Experiment Center (2023070701), and all procedures adhered to the guidelines for the care and use of experimental animals.

### Statistical analysis

ImageJ software was employed for the quantification of protein gray value, cell count, and area. Statistical analyses were conducted utilizing GraphPad Prism (version 8.0.2), SPSS (version 26.0), or R packages. For comparisons between two groups, normally distributed data were analyzed using Student’s t-test, and non-normally distributed data using the Mann-Whitney U test. Comparisons involving more than two groups were conducted using one-way ANOVA. All experiments were replicated three times. *P* < 0.05 represented statistically significant.

## Results

### Comprehensive analysis of SLC2A1 in LUAD

**Fig 1:**
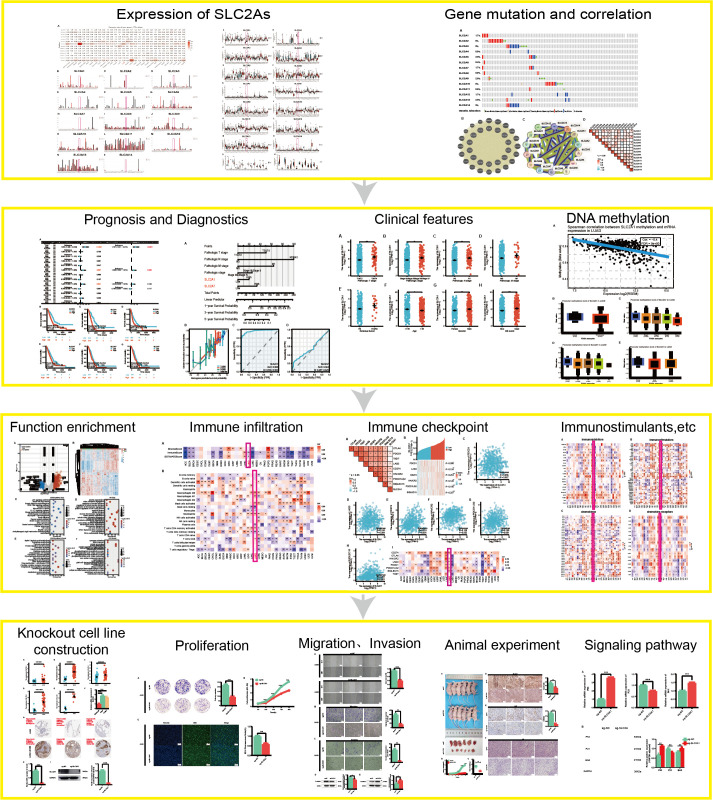
Study flowchart.

This study emphasizes its key insights through graphical summaries, as highlighted in **Fig 1**. The function of *SLC2A1* in lung adenocarcinoma (LUAD) was rigorously examined using an integrated approach, encompassing bioinformatics analysis, as well as *in vivo* and *in vitro* experiments. The findings indicated that elevated levels of *SLC2A1* were closely associated with the malignant progression of LUAD, significantly enhancing tumor cell proliferation, migration, and invasion. These observations point to the potential value of *SLC2A1* as a diagnostic marker, prognostic factor, and therapeutic target in LUAD.

### Differential expression analysis of the *SLC2A* gene family

The GSCA database was deployed to assess the tissue distribution of *SLC2A* family members in normal human tissues and organs. Expression analysis revealed significantly overexpressed *SLC2A1* in nervous tissues compared to other tissues. *SLC2A2* was predominantly expressed in the liver, while *SLC2A3* exhibited significantly higher expression in blood, and muscle tissue displayed high expression of *SLC2A4* (S1A Fig). Furthermore, differential expression profiles for *SLC2A* family members in 31 cancerous tissues alongside their corresponding normal counterparts were obtained using the GEPIA database. Significant overexpression of *SLC2A1*, *SLC2A5*, and *SLC2A11* across various tumor types, including LUAD. In comparison, other family members exhibited lower expression levels in cancerous tissues relative to normal tissues (S1B–S1O Fig).

**Fig 2:**
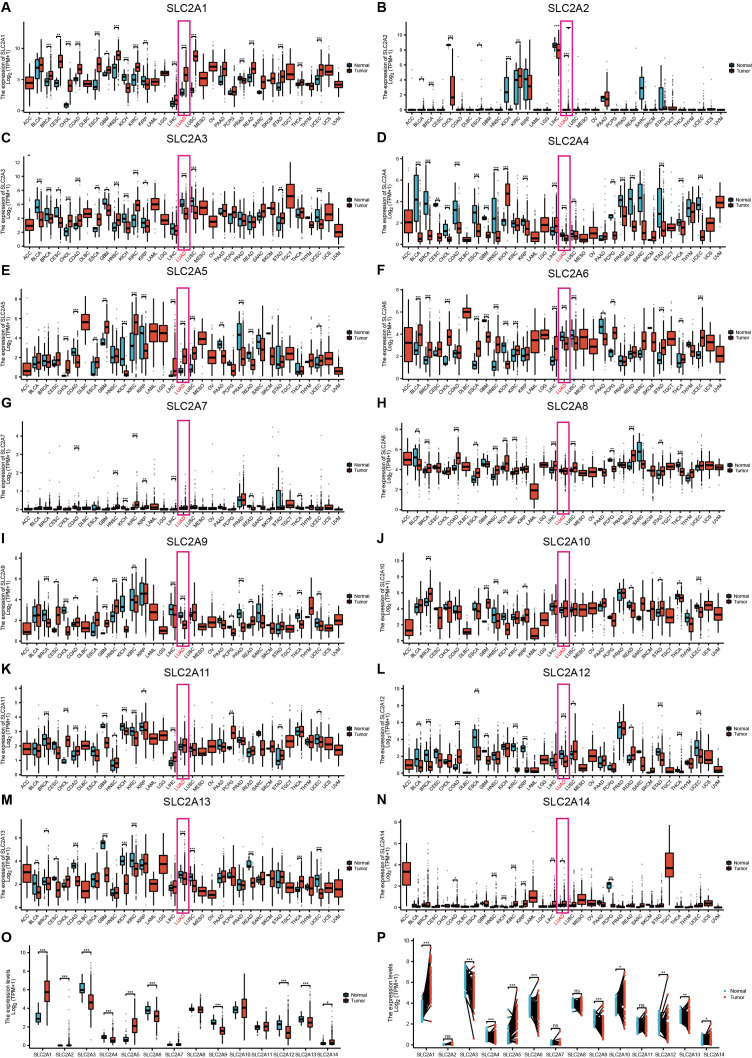
Differential expression of *SLC2A*s in various tumor types. Expression levels of *SLC2A* family members in pancancer tissues: **(A–N)** *SLC2A1*–*14,* as well as in **(O–P)** LUAD and unpaired and paired normal samples, respectively. *: *P* < 0.05, **: *P* < 0.01, ***: *P* < 0.001.

Moreover, significantly upregulated *SLC2A1*/*5/6*/*8*/*14* expression levels were observed in most tumor types among the *SLC2A* family members. Conversely, other family members exhibited significant downregulation or no significant difference in expression levels in most tumor types. Among these, LUAD tissues demonstrated significantly upregulation of *SLC2A1*/*2*/*5*/*14*. In contrast, lower expression levels were observed for *SLC2A3*/*4*/*6*/*12*/*13* (**Fig 2A–2N**). The *SLC2A* family member expression was evaluated in 539 LUAD samples along with 59 non-paired normal samples. Unlike normal tissue, LUAD displayed significantly upregulated *SLC2A1*/*2*/*5*/*14* levels and downregulated *SLC2A3*/*4*/*6*/*9*/*12*/*13* expression (**Fig 2O**). Moreover, significantly upregulated *SLC2A1*/*5*/*10*/*14* levels were observed in 58 LUAD samples and their corresponding normal samples. Conversely, significantly downregulated of *SLC2A3*/*4*/*6*/*9*/*12*/*13* levels were exhibited (**Fig 2P**).

### Gene mutation and interaction analysis

An in-depth analysis of the cBioPortal database revealed a notably low mutation rate within the *SLC2A* gene family in LUAD. The highest observed mutation rate was 6% for *SLC2A3*, followed by 5% for *SLC2A2*/*10* (S2A Fig). Potential interactions between the members of the *SLC2A* gene family and 20 target genes were identified using the GeneMANIA database (S2B Fig). Furthermore, the PPI network demonstrated close correlations among *SLC2A* family members (S2C Fig). Correlation analysis indicated predominantly positive correlations among *SLC2A* family members, with nine positive correlations observed for *SLC2A1* and negative correlations with two other genes (S2D Fig). These findings suggest a potentially pivotal role for *SLC2A1* in cancer development.

### Prognostic value of the *SLC2A* gene family in LUAD

**Fig 3:**
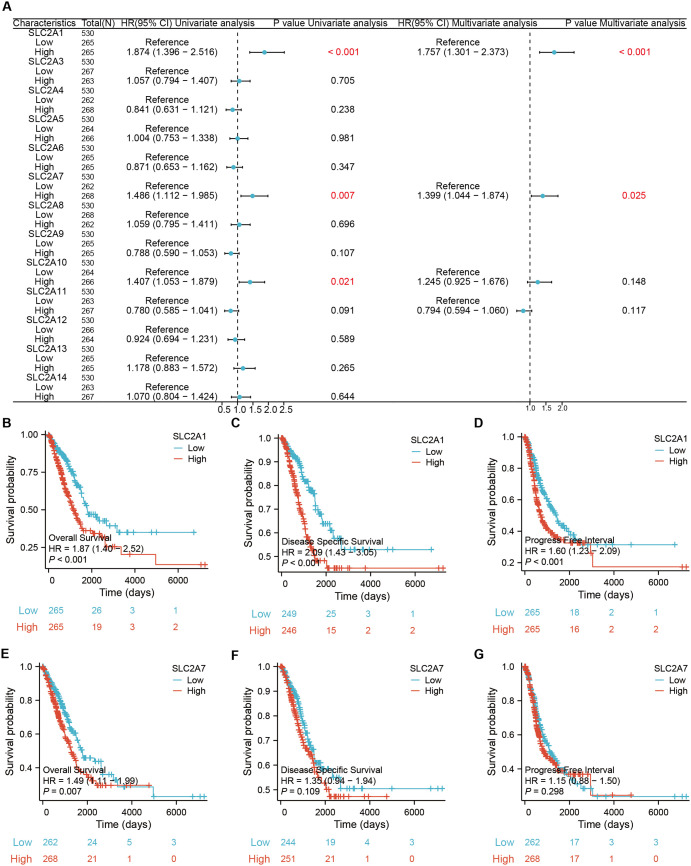
Survival analysis of *SLC2A*s in LUAD. **(A)** Univariate and multivariate regression analyses of *SLC2A* expression in LUAD. **(B–D)** Impact of *SLC2A*1 and **(E–G)** *SLC2A*7 expression on OS, DSS, and DFI in LUAD patients.

Univariate regression analysis revealed a significant correlation between high expression of the *SLC2A1*/*7*/*10* genes and poor prognosis in LUAD patients. Specifically, *SLC2A1* (*P* < 0.001), *SLC2A7* (*P* = 0.007), and *SLC2A10* (*P* = 0.021) were significantly related to poor prognosis (**Fig 3A**). Furthermore, multivariate regression analysis confirmed that upregulated *SLC2A1* (*P* < 0.001) and *SLC2A7* (*P* = 0.025) were independent predictors of poor prognosis in LUAD patients (**Fig 3A**). Kaplan-Meier survival curve analysis manifested that *SLC2A1* upregulation was significantly associated with OS, disease-specific survival (DSS), and progression-free interval (PFI) in LUAD patients (*P* < 0.001) (**Fig 3B–3D**). Additionally, elevated *SLC2A7* levels were identified as a risk factor for OS in LUAD patients (*P* = 0.007) (**Fig 3E–3G**). Overall, these findings suggest *SLC2A1* overexpression and elevated *SLC2A7* levels might serve as potential biomarkers for predicting unfavorable outcomes in LUAD patients.

### *SLC2A1* as a prognostic marker in LUAD

**Fig 4:**
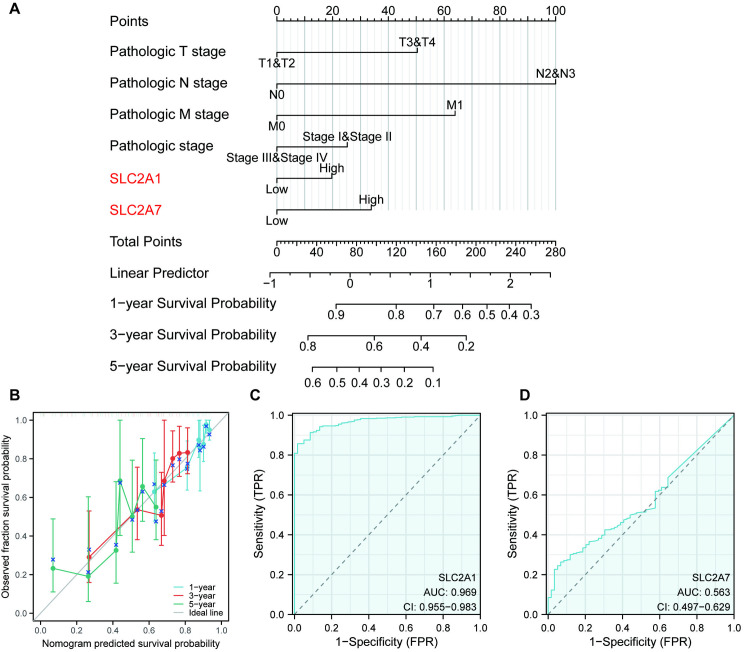
Predictive value of *SLC2A1*/*7* in LUAD. **(A)** The nomogram prediction model of *SLC2A1*/*7* for LUAD. (B) 1-, 3-, and 5-year calibration curves. **(C–D)** Diagnostic ROC curve of *SLC2A1*/*7* in LUAD, respectively.

Considering the pivotal role of *SLC2A1* and SLC2A7 in prognostic prediction for LUAD patients, nomogram prediction models were constructed for these two genes to assess their predictive value. According to the nomogram, a higher score was associated with a worse a poorer prognosis for LUAD patients (**Fig 4A**), providing clinicians with a reference point for evaluating patient outcomes. Calibration curves depicting 1-, 3-, and 5-year survival outcomes demonstrated the high predictive accuracy of the Nomogram model (**Fig 4B**). Furthermore, ROC curves were utilized to evaluate the predictive value of *SLC2A1*/*7* in LUAD. The AUC of *SLC2A1* (0.969) significantly superior to that of *SLC2A7* (0.563) (**Fig 4C and 4D**), suggesting that *SLC2A1* possesses greater diagnostic potential in LUAD and warranting further exploration.

### Association between *SLC2A1* expression and clinicopathological features in LUAD

An analysis of *SLC2A1* expression and the clinicopathological features of LUAD patients was conducted via the Xiantao Academic online tool. Significant overexpression of SLC2A1 was identified in LUAD patients with T3 and T4 tumor stages, N1 nodal involvement, and stage III and IV disease (**P* *< 0.01), whereas no correlation with the M stage and residual tumor was observed (*P* > 0.05) (S3A–S3D Fig). A significantly higher expression of *SLC2A1* was detected in LUAD patients aged ≤ 65 compared to those aged > 65 (*P* < 0.05) (S3F Fig). Moreover, a significant upregulation of *SLC2A1* was predominantly observed in males compared to females (*P* < 0.01) (S3G Fig). Importantly, *SLC2A1* upregulation correlated with a poor OS outcome in LUAD patients (*P* < 0.001) (S3H Fig).

### Methylation analysis of *SLC2A1* in LUAD

The GSCA database was employed to analyze the methylation patterns and mRNA expression of *SLC2A1*. A significant negative correlation was identified between *SLC2A1* expression and methylation level in LUAD was identified (Cor = –0.4, FDR < 0.01) (S4A Fig). Furthermore, the *SLC2A1* methylation levels in normal and LUAD tissues were determined via the UALCAN database (S4B Fig). DNA methylation levels were significantly lower in LUAD than in normal tissues. Furthermore, reduced methylation of *SLC2A1* correlated with clinical parameters of LUAD, including tumor grade, N stage, and sex (S4C–S4E Fig). These findings suggest that decreased methylation level of *SLC2A1* may serve as a potential indicator for assessing clinical characteristics in LUAD.

### Functional enrichment analysis of *SLC2A1* in LUAD

**Fig 5:**
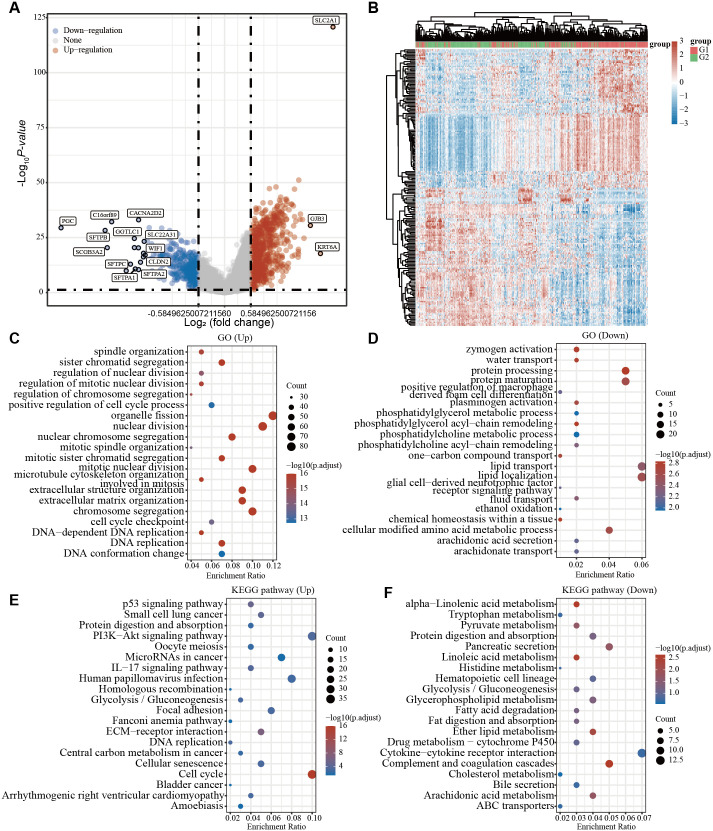
Functional enrichment analysis of *SLC2A1* in LUAD. **(A)** Volcano plot: *SLC2A1* DEGs in LUAD, blue indicates downregulated genes, and red indicates upregulated genes. **(B)** Heat maps: DEG expression levels. **(C–D)** GO enrichment analysis: Upregulated and downregulated genes, respectively. **(E–F)** KEGG enrichment analysis: Upregulated and downregulated genes, respectively. DEGs: Differentially expressed genes.

We previously established that *SLC2A1* was overexpressed in LUAD and was closely linked to poor survival. Nonetheless, the function and probable mechanisms of *SLC2A1* in LUAD remained unclear. GO and KEGG enrichment analyses were performed to explore the functional role of *SLC2A1* in LUAD. Utilizing data from TCGA, we identified 1145 DEGs associated with high and low *SLC2A1* expression levels in LUAD, with 741 upregulated and 404 downregulated (**Fig 5A and 5B**). GO enrichment analysis elucidated that upregulated genes were primarily involved in organelle fission, nuclear division, and DNA replication processes. Conversely, downregulated genes were enriched for protein processing, lipid transport, and localization (**Fig 5C and 5D**). Furthermore, KEGG enrichment analysis demonstrated significant enrichment of upregulated differential genes in the P53, PI3K-Akt, and cell cycle signaling pathways. Downregulated genes were implicated in tryptophan metabolism, cholesterol metabolism, and arachidonic acid metabolism pathways (**Fig 5E and 5F**). These findings suggested that *SLC2A1* might participate in multiple functions within LUAD pathogenesis.

### Correlation analysis of *SLC2A1* with the tumor microenvironment and immune infiltration

The correlation between *SLC2A1* and the Stromal, Immune, and ESTIMATE scores of various tumors, including LUAD, was analyzed to evaluate the relationship between *SLC2A1* and the tumor microenvironment and immune infiltration. A significant negative relationship was demonstrated between most tumors and all three scores, with strong associations observed in ACC and LUSC. Conversely, positive correlations were identified with KIRC and PCPG (S5A Fig). In the case of LUAD, *SLC2A1* expression exhibited a negative correlation with the Immune and ESTIMATE Scores but no correlation with the Stromal Score (S5A Fig). The enrichment analysis results demonstrated a positive correlation between *SLC2A1* expression and M0/M1 macrophages, activated mast cells, Neutrophils, CD4 memory-activated T cells, and CD8 T cells in LUAD (S5B Fig). Conversely, significantly negative correlations with resting mast cells, monocytes, plasma cells, and CD4 memory resting T cells were observed (S5B Fig). These findings strongly suggest a complex relationship between *SLC2A1* expression and immune infiltration across various tumors types, including LUAD.

### Correlation analysis between *SLC2A1* and immune checkpoints in LUAD

The tumor immune response is influenced not only by the immune cell expression levels but also closely linked to the immune checkpoint expression. The correlation between *SLC2A1* expression and eight immune checkpoints (CTLA4, PDCD1, TIGIT, LAG3*,* CD274, HAVCR2, PDCD1LG2, and SIGLEC15) was further investigated across multiple cancer types. A significant positive link between *SLC2A1* expression alongside six immune checkpoints (PDCD1, LAG3, CD274, HAVCR2, PDCD1LG2, and SIGLEC15) was observed in LUAD (S6A Fig). Subsequent analysis showcased significant positive correlations between *SLC2A1* expression in LUAD and all eight immune checkpoint molecules (S6B–S6I Fig).

### Correlation between *SLC2A1* expression and multiple immunomodulators in pancancer

The relation between *SLC2A1* expression and immunostimulants, immunosuppressants, chemokines, and their receptors was further explored utilizing the TISIDB online platform. Results indicated a significant positive correlation between *SLC2A1* and immunostimulants across most cancer types, with CD276 and PVR predominantly implicated in LUAD (S7A Fig). Interestingly, *SLC2A1* expression was also positively associated with immunosuppressants in multiple cancers, with CD274 and TGFBR1 primarily involved in LUAD (S7B Fig). A positive correlation was recognized between *SLC2A1* expression and chemokines in numerous cancer types, with CCL7/26 and CXCL8 being primarily associated with LUAD (S7C Fig). Additionally, *SLC2A1* expression exhibited a predominantly positive correlation with chemokine receptors, although a negative correlation was observed in LUAD, particularly with CCR6/7 (S7D Fig). These findings collectively suggest a pivotal role for *SLC2A1* in immune modulation across various cancer types, including LUAD.

### Expression validation and knockout cell line construction of *SLC2A1* in LUAD

**Fig 6:**
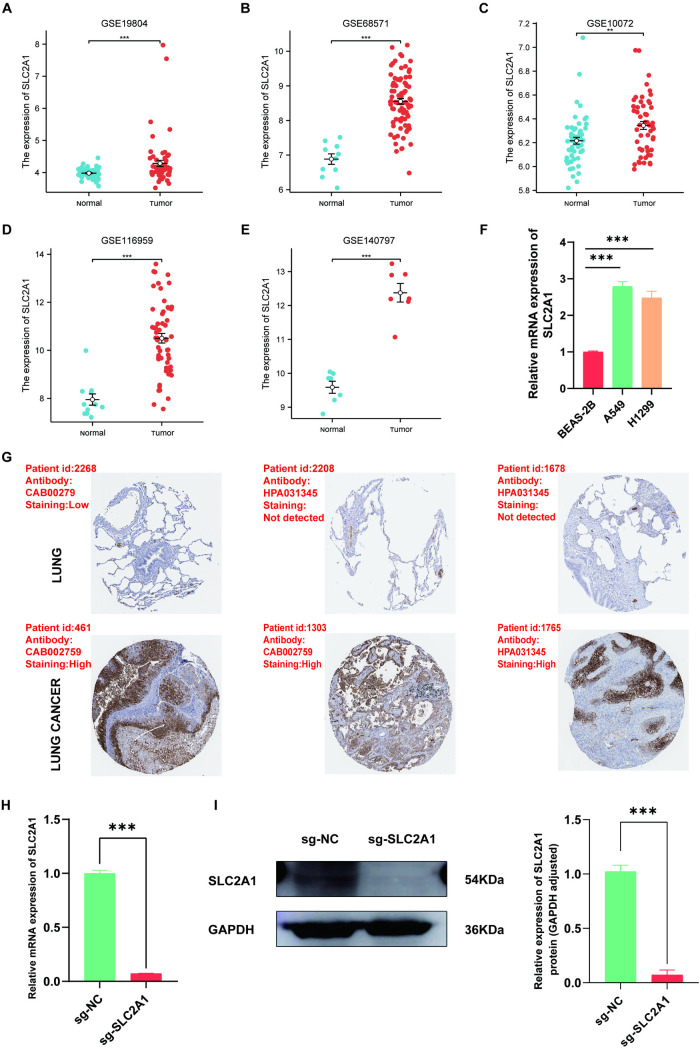
Expression levels of *SLC2A1* in LUAD cells, tissues, and knockout cell line. **(A–E)** *SLC2A1* differential expression in LUAD in GSE19804, GSE6857, GSE10072, GSE116959 and GSE140797 datasets, respectively. **(F)** *SLC2A1* mRNA expression levels in Beas-2B and LUAD cells A549 and H1299. **(G)** Immunohistochemical staining: *SLC2A1* protein expression level in LUAD in the HPA database. (H–I) RT-qPCR and Western blot: validation of *SLC2A1* knockout levels, respectively.

To further assess the impact of abnormal *SLC2A1* expression on the occurrence and progression of LUAD, *SLC2A1* expression was validated across five datasets: GSE19804, GSE68571, GSE10072, GSE116959, and GSE140797. *SLC2A1* exhibited a significant upregulation in cancer tissues in comparison to normal tissues (**P* *< 0.001) (**Fig 6A–6E**). RT-qPCR analysis demonstrated that the *SLC2A1* was significantly overexpressed in LUAD cells compared to Beas-2B and H1299 cells (**P* *< 0.01) (**Fig 6F**). Analysis of the Human Protein Atlas (HPA) database revealed significant upregulation of *SLC2A1* protein levels in LUAD tissues in contrast to normal tissues (**Fig 6G**).

Furthermore, the *SLC2A1* gene knockout cell line (sg-SLC2A1) was successfully constructed in A549 LUAD cells to evaluate the *SLC2A1* function in the LUAD occurrence and development. RT-qPCR and Western Blot analyses affirmed that both mRNA and protein expression levels of *SLC2A1* were virtually undetectable in the knockout cell line (**P* *< 0.001) (**Fig 6H and 6I**). The raw images of WB can be seen in the supporting information file “S1 Data”.

### *SLC2A1* deletion significantly inhibits LUAD cell line A549 proliferation ability

**Fig 7:**
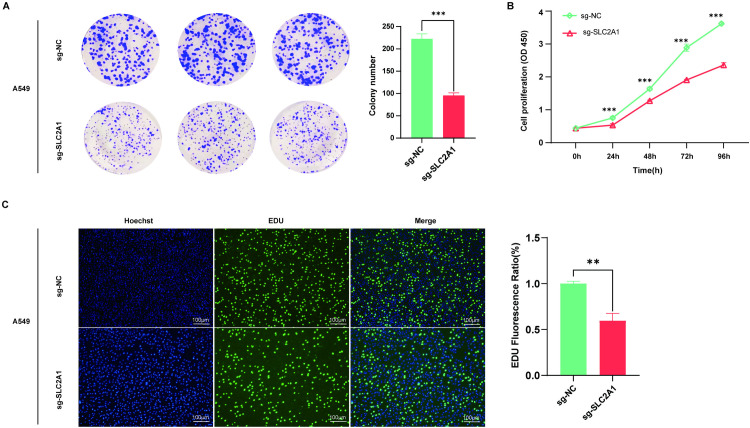
Impact of *SLC2A1* knockout on LUAD cell line A549 proliferation. **(A)** Colony formation experiment. **(B)** CCK-8 assay. **(C)** EdU staining assay.

The impact of *SLC2A1* knockout on A549 cell proliferation was investigated using clony formation, CCK-8, and EdU experiments. The colony formation assays demonstrated a significantly decreased clonogenic capacity in the knockout cell line compared to the wild-type A549 cells (**P* *< 0.001) (**Fig 7A**). Additionally, a significant reduction of proliferative activity was examined in the knockout cell line compared with the wild-type A549 cell line as determined by CCK-8 assay (**P* *< 0.001) (**Fig 7B**). Furthermore, EdU staining experiments indicated that *SLC2A1* knockout significantly suppressed A549 cell proliferation (**P* *< 0.01) (**Fig 7C**). These outcomes suggest that SLC2A1 plays a role in promoting the LUAD cell proliferation of LUAD cells.

### Effect of *SLC2A1* knockout on the migration and invasion ability of LUAD cell line A549

**Fig 8:**
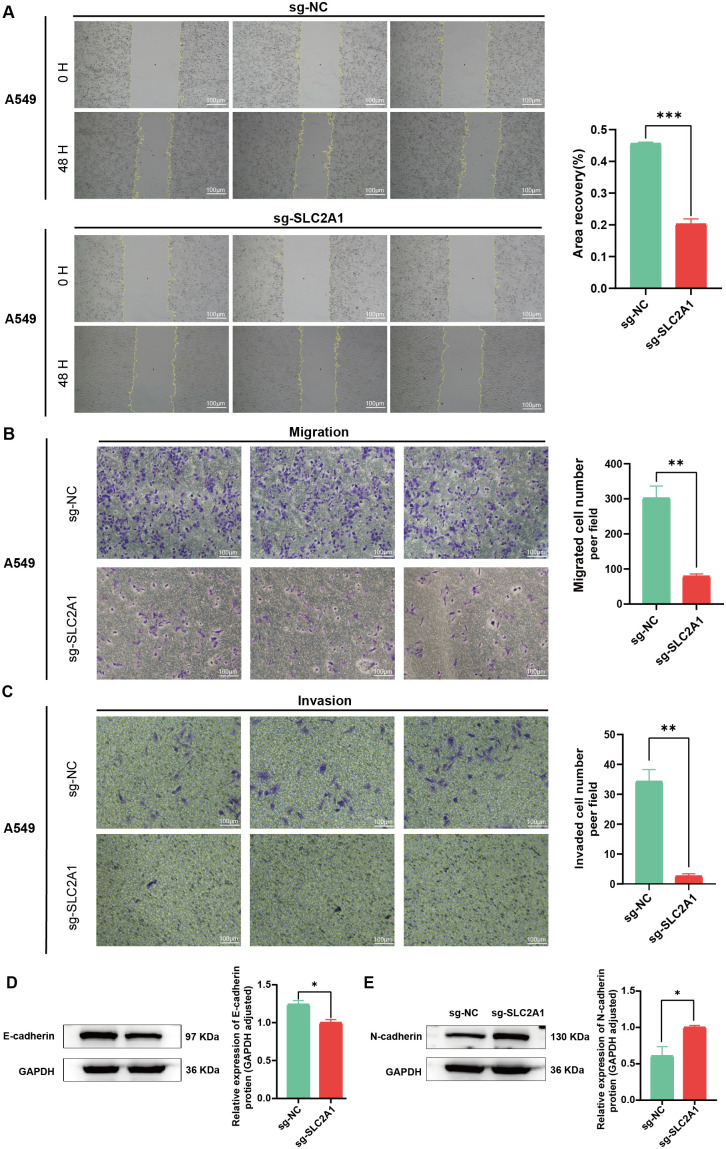
Effect of *SLC2A1* knockout LUAD cell line A549 migration and invasion. **(A)** Wound healing assay: Impact of *SLC2A1* knockout on the migratory capacity of A549 cells. **(B–C)** Transwell migration assay: Effect of *SLC2A1* knockout on A549 cell migratory and invasive potentials, respectively. **(D–E)** E-/N-cadherin protein expression levels in the knockout cell lines, respectively. *P < 0.05, **P < 0.01, ***P < 0.001.

The impact of *SLC2A1* on the A549 cells migratory and invasive capabilities was further investigated using scratch and Transwell assays. A significant reduction in migration ability was observed following *SLC2A1* deletion in the A549 cell line, as demonstrated by the scratch assay (**P* *< 0.001) (**Fig 8A**). The Transwell migration assay similarly revealed a substantial inhibition of cell migration upon *SLC2A1* deletion (**P* *< 0.01) (**Fig 8B**). Moreover, invasion assays conducted using Transwell chambers indicated a significant decrease in invasion ability in the knockout cell line compared to the wild-type control (**P* *< 0.01) (**Fig 8C**). Western Blot analysis of EMT-related protein expression illustrated a significantly downregulated E-cadherin (*P* < 0.05) while a significantly upregulated N-cadherin expression (*P* < 0.05) in the *SLC2A1* knockout cell line (**Figs 8D and 8E**). Collectively, these findings suggest that *SLC2A1* promotes the migration and invasion of LUAD cells.

### Validation of the effect of *SLC2A1* deletion on LUAD pathogenesis in animal experiments

**Fig 9:**
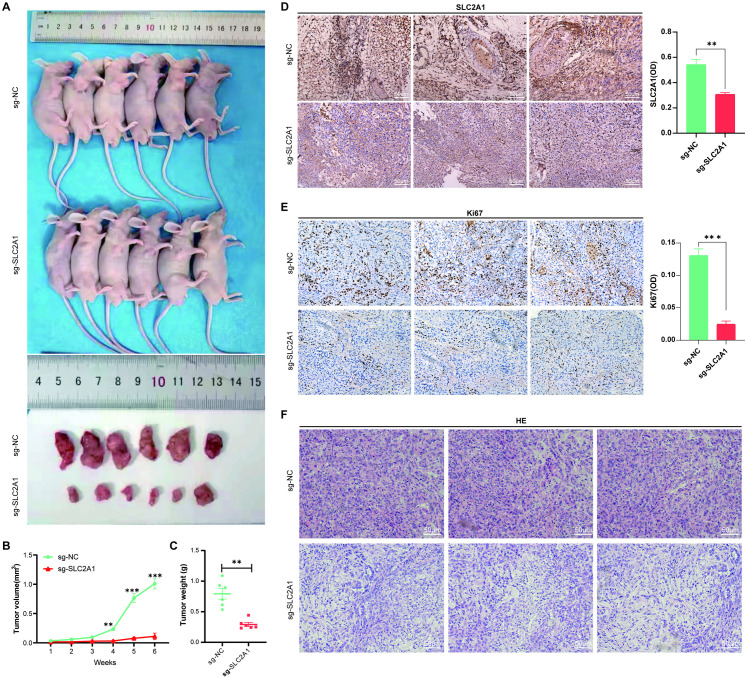
Absence of *SLC2A1* inhibits tumor growth in nude mice. **(A)** Imaging results of tumor-forming nude mice and tumor tissues. **(B)** Changes in tumor tissue volume following 1–6 weeks of tumor cell inoculation. **(C)** Weight of the tumor tissues in nude mice after six weeks of tumor cell inoculation. **(D–F)** Immunohistochemical (IHC) and hematoxylin-eosin (H&E) staining analyses of SLC2A1 expression in the tumor tissues.

The nude mouse tumorigenesis experiments were carried out to verify the *SLC2A1* function in the progression of LUAD. The findings showed a significant suppression of tumor growth in the *SLC2A1*-deficient group compared to the control group, with tumor size and weight being significantly lower (**P* *< 0.01) (**Figs 9A**–**9C**). Furthermore, hematoxylin-eosin (H&E) staining and immunohistochemistry (IHC) analyses revealed a reduced number of mitotic cells and significantly weakened expression of the proliferation marker Ki67 (**P* *< 0.001), and a reduced number of mitotic cells in the *SLC2A1*-deficient group compared to the control group, consistent with the *in vitro* proliferation experiment findings (**Figs 9D**–**9F**). Collectively, *SLC2A1* possesses an important function in the LUAD progression.

### *SLC2A1* may contribute to LUAD progression via the P53 signaling pathway

**Fig 10:**
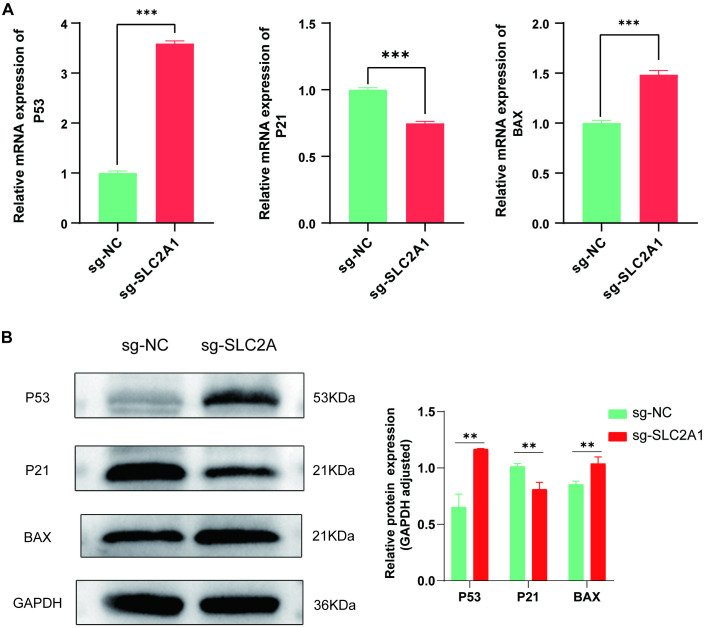
Analysis of P53 signaling pathway in LUAD after *SLC2A1* knockout. (A) RT-qPCR results: key molecules P53, P21, and BAX expression levels in the *SLC2A1* knockout cell line, respectively. (B) WB results: protein expression levels of P53, P21, and BAX in the *SLC2A1* knockout cell line, respectively.

Based on the preceding GO/KEGG enrichment analysis results, the differentially expressed genes of associated with *SLC2A1* were found to be significantly enriched in the P53 pathway in LUAD. Given the pivotal role of the P53 signaling pathway in tumorigenesis, RT-qPCR and WB experiments were conducted to assess the expression of the key molecules within this pathway. The results indicated that, compared to the wild-type cell line, the expression of the key molecule P53 was significantly upregulated in the sg-*SLC2A1* knockout cell line, while P21 expression was significantly downregulated, and BAX expression was significantly upregulated (**Fig 10A and 10B**). The raw images of WB can be found in the supporting information file “S1 Data”. However, the precise mechanism underlying these changes requires further exploration.

## Discussion

Tumor proliferation necessitates substantial energy and nutrient supply, largely sustained through the oxidative metabolism of glucose. Regulation of glucose uptake and utilization plays a critical role in influencing tumor cell growth, providing promising opportunities for the development of innovative cancer therapies. The GLUT protein, encoded by the *SLC2A* genes family, is instrumental in facilitating glucose uptake [6]. Abnormal GLUT expression and activity in tumor cells often lead to enhanced glucose absorption, a metabolic adaptation known recognized as the Warburg effect. The elevated expression of *SLC2As* has been found in various cancers, including leukemia, papillary thyroid carcinoma, and LUAD [6,25,26]. Moreover, *SLC2A* expression can impact tumor cell aggressiveness, thereby affecting tumor growth and metastasis [27–30]. Consequently, modulating *SLC2A* expression and function may offer promising strategies for cancer therapy and prognosis. However, the role of the *SLC2A* gene family in LUAD remains largely unexplored. Herein, a comprehensive multi-omics analysis was conducted to address this gap and investigate *SLC2A* expression levels, mutations, diagnostic value, prognostic significance, and immune relevance in LUAD tissues. Besides, differential expression analysis between cancerous and adjacent normal tissues was carried out with emphasis on the *SLC2A1* gene. The impact of *SLC2A1* knockout on malignant biological behaviors associated with LUAD was also systematically evaluated.

We first performed differential expression analysis and then examined the clinical significance of *SLC2A*s in LUAD. In the TCGA-LUAD dataset, in comparison to normal tissues, *SLC2A1*/*5*/*14* expression levels were found to be upregulated in LUAD, while levels of *SLC2A3*/*4*/*6*/*9*/*12*/*13* were downregulated. Previous studies have indicated a relationship between genetic alterations in *SLC2A*s and certain disease risks [8]. Subsequently, the genetic alterations of *SLC2A*s in LUAD were assessed. The findings revealed that mutations in *SLC2A*s within LUAD were relatively infrequent, with the highest observed mutation rate of 6%). Based on these observations, it was inferred that *SLC2A*s exhibited high conservation in LUAD, and their mutations had a relatively limited influence on disease development. Numerous studies have unequivocally demonstrated the potential of *SLC2A*s as promising biological targets for tumor diagnosis and prognosis. For instance, increased levels of *SLC2A1* have been considered a potential prognostic marker for LUAD, colorectal cancer, and breast cancer [11,31,32]. Similarly, *SLC2A7* has been identified as both a prognostic marker and a novel immunotherapy target for gastric cancer [33]. Furthermore, *SLC2A3* has been shown to influence colorectal cancer progression and head and neck squamous cell carcinoma by governing Epithelial-Mesenchymal Transition (EMT) and immune responses [27,34]. Building upon these findings, an investigation of the *SLC2A1* prognostic and diagnostic implications in LUAD patients was conducted. Both univariate and multivariate regression analyses consistently indicated that elevated levels of both *SLC2A1*/*7* were correlated with decreased OS rates among individuals with LUAD. Moreover, increased *SLC2A1* expression was associated with a notable reduction in both DSS and DFI, while no such association was observed for increased *SLC2A7* expression. These results suggested that both *SLC2A1/7* may be potential prognostic biomarkers for LUAD. Moreover, the ROC curve analysis indicated that *SLC2A1* presented a higher diagnostic value than *SLC2A7* in LUAD, suggesting that *SLC2A1* may serve as a more suitable diagnostic marker for LUAD. Therefore, this study further focused on investigating the *SLC2A1* function in LUAD development.

To explore the role of *SLC2A1* in LUAD development, an analysis of the relationship between *SLC2A1* expression, along with clinicopathological features and DNA methylation, was conducted. High expression of *SLC2A1* was recognized in advanced clinical stages of LUAD patients. Additionally, correlations between SLC2A1 expression and factors such as age, gender (male), and OS were identified, consistent with previous research [12]. DNA methylation, recognized as an epigenetic mechanism, exerts a promotional effect on various processes implicated in tumor formation [35]. Hypomethylation has been proposed as a trigger for cancer cell transcription, initiating tumor formation [36]. Previous studies have established a relationship between abnormal methylation of LUAD and *SLC2A1* expression [37]. A negative correlation between SLC2A1 mRNA expression and DNA methylation was identified in this study. Furthermore, LUAD tissues showed significantly lower *SLC2A1* promoter methylation levels than normal tissues, a trend that persisted across LUAD grading, N stage, and gender. Consequently, it can be inferred that the regulation of *SLC2A1* expression in LUAD may influenced by DNA methylation mechanisms, subsequently contributing to tumor progression and prognosis. The differential expression analysis was performed to elucidate the biological function of *SLC2A1* in LUAD, followed by GO/KEGG enrichment analysis. The upregulated DEGs were significantly enriched in DNA replication, the P53, and the PI3K-Akt signaling pathways, suggesting their potential involvement in intracellular signal transduction within tumor cells. Conversely, the downregulated genes were primarily associated with protein processing, tryptophan metabolism, and cholesterol metabolism, highlighting their significance in regulating tumor cell metabolism. Dysregulation of the P53 signaling pathway is commonly observed in tumor development and can influence the immune response within tumors [38]. The PI3K/Akt pathway possesses a critical function in regulating the malignant behavior of tumor cells and modulating the immune cell involvement in tumor formation [39]. In conclusion, *SLC2A1* may modulate the development and progression of LUAD through multiple pathways.

Recently, there has been a substantial increase in research focused on the immune microenvironment, with emphasis on the role of immune checkpoint molecules within the tumor microenvironment. This study delved further into the correlation between *SLC2A1* expression and the tumor microenvironment. Our findings indicated a significant elevation in the infiltration proportion of activated mast cells, activated CD4 memory T cells, and CD8^+^ T cells associated with high *SLC2A1* expression. Conversely, a significant reduction in the resting mast cells and resting CD4 memory T cells abundance was observed. Previous studies have linked activated mast cells, activated CD4 memory T cells, and CD8^+^ T cells to poor prognosis across various cancer types [40–43]. Our analysis of immune checkpoints revealed a significant positive association between *SLC2A1* high expression and six immune checkpoint molecules in LUAD: PDCD1, LAG3, CD274 (PD-L1), HAVCR2 (TIM-3), PDCD1LG2 (PD-L2), and SIGLEC15. High *SLC2A1* expression and upregulation of certain immune checkpoints have been proposed to be correlated in LUAD [12]. Moreover, *SLC2A1* demonstrated significant positive correlations with immune regulatory factors, particularly CD276 (B7-H3), PVR (CD155), CD274 (PD-L1), TGFBR1 (TGF-beta receptor 1), CCL7 (MCP-3), CCL26 (Eotaxin-3), and CXCL8 (IL-8). Conversely, a significant negative correlation with CCL6 was found. These outcomes collectively suggest that *SLC2A1* possesses a critical function in modulating the immune microenvironment within LUAD and may present a promising immunotherapeutic target for this disease.

Subsequent analysis revealed significantly elevated levels of both SLC2A1 mRNA and protein in LUAD tissues compared to normal tissues. Moreover, the A549 cell line exhibited higher *SLC2A1* expression than the H1299 cell line. To elucidate the biological function of *SLC2A*1, *in vitro* and *in vivo* studies were conducted employing the A549 cell line. Previous research has established that increased *SLC2A1* expression enhances the aggressiveness of hepatocellular carcinoma, LUAD, and colorectal cancer cells [44–46]. Deletion of the *SLC2A1* gene in the A549 human LUAD cell line significantly inhibited proliferation compared to wild-type cells, highlighting the crucial function of *SLC2A1* in the malignant biological processes of LUAD. E-cadherin expression is known to inhibit tumor cell migration and invasion, while N-cadherin expression promotes these processes [47,48]. Following *SLC2A1* knockout in A549 cells, E-cadherin displayed a significant downregulation, while N-cadherin exhibited upregulation, consistent with previous findings in breast cancer [49]. To further investigate *SLC2A1*’s role in LUAD development, a nude mouse tumorigenesis experiment was performed. Deletion of *SLC2A1* significantly inhibited tumor growth compared to the control, accompanied by a significant reduction in Ki67 expression and cellular proliferation. These findings collectively suggest that elevated *SLC2A1* levels promote malignant tumor progression both *in vitro* and *in vivo*.

Given the significant involvement of *SLC2A1* in LUAD pathogenesis, a subsequent investigation was performed to explore the signaling pathways potentially modulated by *SLC2A1* during LUAD progression. Gene functional enrichment analysis manifested that *SLC2A1*-related DEGs were enriched in P53 signaling. The P53 pathway is a critical apoptotic target across various tumor types, capable of suppressing key dysregulated pathways in tumors while inhibiting tumor proliferation and metastasis [37]. To further elucidate the impact of *SLC2A1* on this pathway, the expression of key molecules within the P53 pathway, including P53, P21, and BAX, was examined following *SLC2A1* deletion [50–52]. A negative relation was found between *SLC2A1* expression and both P53 and BAX, while a positive correlation with P21 was identified. Considering P21’s dual role as an anti-cancer factor with potential pro-cancer effects linked to tumor proliferation, migration, and invasion, leading to unfavorable patient prognosis, it is highly conceivable that *SLC2A1* may modulate LUAD proliferation, migration, invasion, and apoptosis through the P53 signaling pathway, thereby influencing LUAD prognosis.

## Conclusion

*SLC2A* family genes exhibit elevated expression in various tumor tissues, including LUAD, and are linked to poor patient prognosis. *SLC2A1*, in particular, holds significant potential as a prognostic biomarker for the LUAD patient’s diagnosis and prognosis. The absence of *SLC2A1* markedly inhibits A549 cell proliferation, migration, and invasion, while *SLC2A1* knockout significantly suppresses tumor formation in nude mice. These findings collectively illustrate that *SLC2A1* represents a promising biomarker and therapeutic target for LUAD.

## Supporting information

S1 Fig Expression of *SLC2A*s in human normal tissues and tumor tissues. (A) Heat map: SLC2A expression in human normal tissues and organs in the GSCA database: (B–K) expression profile of *SLC2A1–10* and (L–O) median expression levels of *SLC2A11–14*.
(TIF)

S2 Fig Gene mutation and protein-protein interaction analysis of *SLC2A*s. (A) Genetic variation of the *SLC2A* family in LUAD based on the cBioPortal database. (B–C) PPI network of *SLC2A* family genes by GeneMANIA and STRING databases. (D) Correlation analysis among *SLC2A* family proteins in LUAD based on the Xiantao Academic database. LUAD: Lung adenocarcinoma. PPI: Protein-protein interaction.
(TIF)

S3 Fig Correlation analysis between *SLC2A1* expression and clinicopathological features of LUAD patients. (A) Pathological T stage. (B) Pathologic stage. (C) Pathologic N stage. (D) Pathologic M stage. (E) Residual tumor. (F) Age. (G) Gender. (H) OS events.
(TIF)

S4 Fig Methylation of *SLC2A1* in LUAD. (A) Correlation between *SLC2A1* methylation in LUAD and *SLC2A1* mRNA expression. (B) Methylation levels of the *SLC2A1* gene in LUAD tissues. (C) Correlation between the stage of LUAD patients and methylation status of the *SLC2A1* gene. (D) Correlation between N stage and *SLC2A1* methylation in LUAD patients. (E) Relation between gender-specific methylation patterns and expression levels of *SLC2A1* in LUAD patients.
(TIF)

S5 Fig Tumor microenvironment and immune infiltration analysis of *SLC2A1* in Pancancer. (A) Correlation between *SLC2A1* expression and tumor microenvironment and (B) immune cell infiltration in various tumors. *P < 0.05; **P < 0.01; ***P < 0.001.
(TIF)

S6 Fig Correlation between *SLC2A1* expression and immune checkpoints in LUAD. (A) Correlation between *SLC2A1* expression and immune checkpoint in 33 tumors. (B) Heat maps: Correlation between *SLC2A1* expression and immune checkpoint. (C) Correlation of *SLC2A1* expression with PDCD1, LAG3, CD274, HAVCR2, PDCD1LG2 and SIGLEC15; Scatter plots: Positively correlation of *SLC2A1* expression with PDCD1 (D), LAG3 (E), CD274 (F), HAVCR2 (G), PDCD1LG2 (H) and SIGLEC15 (I).
(TIF)

S7 Fig Correlation between *SLC2A1* with immunosuppressants, immunostimulants, chemokines, and chemokine receptors analyzed by TISIDB. (A) Heat map: Correlation analysis between *SLC2A1* expression and immunosuppressive agents and (B) immunostimulants. (C) Heat map: Correlation analysis between *SLC2A1* expression and chemokines and (D) chemokine receptors.
(TIF)

S1 Data Raw_images. (PDF)

## Abbreviations

ATP: Adenosine triphosphate
CCK8: Cell counting kit-8
DMEM: Dulbecco’s modified eagle medium
DSS: Disease-specific Survival
DEGs: Differentially expressed genes
EdU: 5-Ethynyl-2’-deoxyuridine
EMT: Epithelial-Mesenchymal Transition
GAPDH: Glyceraldehyde-3-phosphate dehydrogenase
GEO: Gene expression omnibus
GEPIA: Gene Expression Profiling Interactive Analysis
GLUT1: Glucose transporter 1
GSCA: Gene Set Cancer Analysis
GO: Gene ontology
KEGG: Kyoto Encyclopedia of Genes and Genomes
LUAD: Lung adenocarcinoma
OS: Overall Survival
PFI: Progress Free Interval
PBS: Phosphate-buffered saline
PPI: Protein-protein interaction
ROC: Receiver operating characteristic curve
RT-qPCR: Quantitative real-time polymerase chain reaction
TCGA: The cancer genome atlas.

## References

[1] Jiang F, Liang M, Huang X, Shi W, Wang Y. High expression of PIMREG predicts poor survival outcomes and is correlated with immune infiltrates in lung adenocarcinoma. PeerJ. 2021;9:e11697. doi: 10.7717/peerj.11697 34268011 PMC8269662

[2] Bray F, Laversanne M, Sung H, Ferlay J, Siegel RL, Soerjomataram I, et al. Global cancer statistics 2022: GLOBOCAN estimates of incidence and mortality worldwide for 36 cancers in 185 countries. CA Cancer J Clin. 2024;74(3):229–63. doi: 10.3322/caac.21834 38572751

[3] Song Y, Kelava L, Kiss I. MiRNAs in lung adenocarcinoma: role, diagnosis, prognosis, and therapy. Int J Mol Sci. 2023;24(17):13302. doi: 10.3390/ijms241713302 37686110 PMC10487838

[4] Xia H, Zhang Z, Yuan J, Niu Q. The lncRNA PVT1 promotes invasive growth of lung adenocarcinoma cells by targeting miR-378c to regulate SLC2A1 expression. Hum Cell. 2021;34(1):201–10. doi: 10.1007/s13577-020-00434-7 32960438

[5] Guo W, Sun S, Guo L, Song P, Xue X, Zhang H, et al. Elevated SLC2A1 expression correlates with poor prognosis in patients with surgically resected lung adenocarcinoma: a study based on immunohistochemical analysis and bioinformatics. DNA Cell Biol. 2020;39(4):631–44. doi: 10.1089/dna.2019.5291 32096653

[6] Tilekar K, Upadhyay N, Schweipert M, Hess JD, Macias LH, Mrowka P, et al. Permuted 2,4-thiazolidinedione (TZD) analogs as GLUT inhibitors and their in-vitro evaluation in leukemic cells. Eur J Pharm Sci. 2020;154:105512. doi: 10.1016/j.ejps.2020.105512 32801003 PMC9398548

[7] Mueckler M, Thorens B. The SLC2 (GLUT) family of membrane transporters. Mol Aspects Med. 2013;34(2–3):121–38. doi: 10.1016/j.mam.2012.07.001 23506862 PMC4104978

[8] Holman GD. Structure, function and regulation of mammalian glucose transporters of the SLC2 family. Pflugers Arch. 2020;472(9):1155–75. doi: 10.1007/s00424-020-02411-3 32591905 PMC7462842

[9] Oda Y, Aishima S, Shindo K, Fujino M, Mizuuchi Y, Hattori M, et al. SLC2A1/GLUT1 expression in mural nodules of intraductal papillary mucinous neoplasm of the pancreas. Hum Pathol. 2017;65:71–8. doi: 10.1016/j.humpath.2017.03.008 28412205

[10] Min K-W, Kim D-H, Son BK, Moon KM, Kim SM, Intazur Rahaman M, et al. High SLC2A1 expression associated with suppressing CD8 T cells and B cells promoted cancer survival in gastric cancer. PLoS One. 2021;16(3):e0245075. doi: 10.1371/journal.pone.0245075 33735188 PMC7971512

[11] Liu X-S, Yang J-W, Zeng J, Chen X-Q, Gao Y, Kui X-Y, et al. SLC2A1 is a diagnostic biomarker involved in immune infiltration of colorectal cancer and associated with m6A modification and ceRNA. Front Cell Dev Biol. 2022;10:853596. doi: 10.3389/fcell.2022.853596 35399515 PMC8987357

[12] Wang Y, Wen H, Sun D. SLC2A1 plays a significant prognostic role in lung adenocarcinoma and is associated with tumor immunity based on bioinformatics analysis. Ann Transl Med. 2022;10(9):519. doi: 10.21037/atm-22-1430 35928739 PMC9347052

[13] Lu T-P, Tsai M-H, Lee J-M, Hsu C-P, Chen P-C, Lin C-W, et al. Identification of a novel biomarker, SEMA5A, for non-small cell lung carcinoma in nonsmoking women. Cancer Epidemiol Biomarkers Prev. 2010;19(10):2590–7. doi: 10.1158/1055-9965.EPI-10-0332 20802022

[14] Beer DG, Kardia SLR, Huang C-C, Giordano TJ, Levin AM, Misek DE, et al. Gene-expression profiles predict survival of patients with lung adenocarcinoma. Nat Med. 2002;8(8):816–24. doi: 10.1038/nm733 12118244

[15] Landi MT, Dracheva T, Rotunno M, Figueroa JD, Liu H, Dasgupta A, et al. Gene expression signature of cigarette smoking and its role in lung adenocarcinoma development and survival. PLoS One. 2008;3(2):e1651. doi: 10.1371/journal.pone.0001651 18297132 PMC2249927

[16] Moreno Leon L, Gautier M, Allan R, Ilié M, Nottet N, Pons N, et al. The nuclear hypoxia-regulated NLUCAT1 long non-coding RNA contributes to an aggressive phenotype in lung adenocarcinoma through regulation of oxidative stress. Oncogene. 2019;38(46):7146–65. doi: 10.1038/s41388-019-0935-y 31417181

[17] Liang J, Li H, Han J, Jiang J, Wang J, Li Y, et al. Mex3a interacts with LAMA2 to promote lung adenocarcinoma metastasis via PI3K/AKT pathway. Cell Death Dis. 2020;11(8):614. doi: 10.1038/s41419-020-02858-3 32792503 PMC7427100

[18] Liu C-J, Hu F-F, Xia M-X, Han L, Zhang Q, Guo A-Y. GSCALite: a web server for gene set cancer analysis. Bioinformatics. 2018;34(21):3771–2. doi: 10.1093/bioinformatics/bty411 29790900

[19] Tang Z, Li C, Kang B, Gao G, Li C, Zhang Z. GEPIA: a web server for cancer and normal gene expression profiling and interactive analyses. Nucleic Acids Res. 2017;45(W1):W98–102. doi: 10.1093/nar/gkx247 28407145 PMC5570223

[20] Cerami E, Gao J, Dogrusoz U, Gross BE, Sumer SO, Aksoy BA, et al. The cBio cancer genomics portal: an open platform for exploring multidimensional cancer genomics data. Cancer Discov. 2012;2(5):401–4. doi: 10.1158/2159-8290.CD-12-0095 22588877 PMC3956037

[21] Franz M, Rodriguez H, Lopes C, Zuberi K, Montojo J, Bader GD, et al. GeneMANIA update 2018. Nucleic Acids Res. 2018;46(W1):W60–4. doi: 10.1093/nar/gky311 29912392 PMC6030815

[22] Szklarczyk D, Gable AL, Nastou KC, Lyon D, Kirsch R, Pyysalo S, et al. The STRING database in 2021: customizable protein-protein networks, and functional characterization of user-uploaded gene/measurement sets. Nucleic Acids Res. 2021;49(D1):D605–12. doi: 10.1093/nar/gkaa1074 33237311 PMC7779004

[23] Chandrashekar DS, Karthikeyan SK, Korla PK, Patel H, Shovon AR, Athar M, et al. UALCAN: An update to the integrated cancer data analysis platform. Neoplasia. 2022;25:18–27. doi: 10.1016/j.neo.2022.01.001 35078134 PMC8788199

[24] Ru B, Wong CN, Tong Y, Zhong JY, Zhong SSW, Wu WC, et al. TISIDB: an integrated repository portal for tumor-immune system interactions. Bioinformatics. 2019;35(20):4200–2. doi: 10.1093/bioinformatics/btz210 30903160

[25] Chai YJ, Yi JW, Oh SW, Kim YA, Yi KH, Kim JH, et al. Upregulation of SLC2 (GLUT) family genes is related to poor survival outcomes in papillary thyroid carcinoma: Analysis of data from The Cancer Genome Atlas. Surgery. 2017;161(1):188–94. doi: 10.1016/j.surg.2016.04.050 27842912

[26] Zhang Y, Qin H, Bian J, Ma Z, Yi H. SLC2As as diagnostic markers and therapeutic targets in LUAD patients through bioinformatic analysis. Front Pharmacol. 2022;13:1045179. doi: 10.3389/fphar.2022.1045179 36518662 PMC9742449

[27] Chai F, Zhang J, Fu T, Jiang P, Huang Y, Wang L, et al. Identification of SLC2A3 as a prognostic indicator correlated with the NF-κB/EMT axis and immune response in head and neck squamous cell carcinoma. Channels (Austin). 2023;17(1):2208928. doi: 10.1080/19336950.2023.2208928 37134043 PMC10158547

[28] Weng Y, Fan X, Bai Y, Wang S, Huang H, Yang H, et al. SLC2A5 promotes lung adenocarcinoma cell growth and metastasis by enhancing fructose utilization. Cell Death Discov. 2018;4:38. doi: 10.1038/s41420-018-0038-5 29531835 PMC5841403

[29] Lin M, Fang Y, Li Z, Li Y, Feng X, Zhan Y, et al. S100P contributes to promoter demethylation and transcriptional activation of SLC2A5 to promote metastasis in colorectal cancer. Br J Cancer. 2021;125(5):734–47. doi: 10.1038/s41416-021-01306-z 34188196 PMC8405647

[30] Han X, Yang J, Li D, Guo Z. Overexpression of uric acid transporter SLC2A9 inhibits proliferation of hepatocellular carcinoma cells. Oncol Res. 2019;27(5):533–40. doi: 10.3727/096504018X15199489058224 29523220 PMC7848443

[31] Okcu O, Sen B, Ozturk C, Guvendi GF, Bedir R. GLUT-1 expression in breast cancer. Turk Patoloji Derg. 2022;38(2):114–21. doi: 10.5146/tjpath.2021.01557 34580846 PMC9999698

[32] Ancey P-B, Contat C, Boivin G, Sabatino S, Pascual J, Zangger N, et al. GLUT1 expression in tumor-associated neutrophils promotes lung cancer growth and resistance to radiotherapy. Cancer Res. 2021;81(9):2345–57. doi: 10.1158/0008-5472.CAN-20-2870 33753374 PMC8137580

[33] Zhang W, Zhou D, Song S, Hong X, Xu Y, Wu Y, et al. Prediction and verification of the prognostic biomarker SLC2A2 and its association with immune infiltration in gastric cancer. Oncol Lett. 2023;27(2):70. doi: 10.3892/ol.2023.14203 38192676 PMC10773219

[34] Yao X, He Z, Qin C, Deng X, Bai L, Li G, et al. SLC2A3 promotes macrophage infiltration by glycolysis reprogramming in gastric cancer. Cancer Cell Int. 2020;20:503. doi: 10.1186/s12935-020-01599-9 33061855 PMC7552479

[35] Gallego-Bartolomé J. DNA methylation in plants: mechanisms and tools for targeted manipulation. New Phytol. 2020;227(1):38–44. doi: 10.1111/nph.16529 32159848

[36] Mangelinck A, Mann C. DNA methylation and histone variants in aging and cancer. Int Rev Cell Mol Biol. 2021;364:1–110. doi: 10.1016/bs.ircmb.2021.06.002 34507780

[37] Zheng H, Long G, Zheng Y, Yang X, Cai W, He S, et al. Glycolysis-related SLC2A1 is a potential pan-cancer biomarker for prognosis and immunotherapy. Cancers (Basel). 2022;14(21):5344. doi: 10.3390/cancers14215344 36358765 PMC9657346

[38] Zhang D, Zou T, Liu Q, Chen J, Xiao M, Zheng A, et al. Transcriptomic characterization revealed that METTL7A inhibits melanoma progression via the p53 signaling pathway and immunomodulatory pathway. PeerJ. 2023;11:e15799. doi: 10.7717/peerj.15799 37547717 PMC10404031

[39] Yu L, Wei J, Liu P. Attacking the PI3K/Akt/mTOR signaling pathway for targeted therapeutic treatment in human cancer. Semin Cancer Biol. 2022;85:69–94. doi: 10.1016/j.semcancer.2021.06.019 34175443

[40] Sun Y, Liu L, Fu Y, Liu Y, Gao X, Xia X, et al. Metabolic reprogramming involves in transition of activated/resting CD4+ memory T cells and prognosis of gastric cancer. Front Immunol. 2023;14:1275461. doi: 10.3389/fimmu.2023.1275461 38090588 PMC10711070

[41] Gadi D, Griffith A, Tyekucheva S, Wang Z, Rai V, Vartanov A, et al. A T cell inflammatory phenotype is associated with autoimmune toxicity of the PI3K inhibitor duvelisib in chronic lymphocytic leukemia. Leukemia. 2022;36(3):723–32. doi: 10.1038/s41375-021-01441-9 34743191 PMC8891037

[42] Fan F, Gao J, Zhao Y, Wang J, Meng L, Ma J, et al. Elevated mast cell abundance is associated with enrichment of CCR2+ Cytotoxic T cells and favorable prognosis in lung adenocarcinoma. Cancer Res. 2023;83(16):2690–703. doi: 10.1158/0008-5472.CAN-22-3140 37249584 PMC10425735

[43] Fan L, Ru J, Liu T, Ma C. Identification of a novel prognostic gene signature from the immune cell infiltration landscape of osteosarcoma. Front Cell Dev Biol. 2021;9:718624. doi: 10.3389/fcell.2021.718624 34552929 PMC8450587

[44] Wang Y, Shi S, Ding Y, Wang Z, Liu S, Yang J, et al. Metabolic reprogramming induced by inhibition of SLC2A1 suppresses tumor progression in lung adenocarcinoma. Int J Clin Exp Pathol. 2017;10(11):10759–69. 31966419 PMC6965847

[45] Li B, Kang H, Xiao Y, Du Y, Xiao Y, Song G, et al. LncRNA GAL promotes colorectal cancer liver metastasis through stabilizing GLUT1. Oncogene. 2022;41(13):1882–94. doi: 10.1038/s41388-022-02230-z 35149838

[46] Fang X, Liu Y, Xiao W, Zhao N, Zhu C, Yu D, et al. Prognostic SLC family genes promote cell proliferation, migration, and invasion in hepatocellular carcinoma. Acta Biochim Biophys Sin (Shanghai). 2021;53(8):1065–75. doi: 10.1093/abbs/gmab076 34128989

[47] Na T-Y, Schecterson L, Mendonsa AM, Gumbiner BM. The functional activity of E-cadherin controls tumor cell metastasis at multiple steps. Proc Natl Acad Sci U S A. 2020;117(11):5931–7. doi: 10.1073/pnas.1918167117 32127478 PMC7084067

[48] Cao Z-Q, Wang Z, Leng P. Aberrant N-cadherin expression in cancer. Biomed Pharmacother. 2019;118:109320. doi: 10.1016/j.biopha.2019.109320 31545265

[49] Oh S, Kim H, Nam K, Shin I. Glut1 promotes cell proliferation, migration and invasion by regulating epidermal growth factor receptor and integrin signaling in triple-negative breast cancer cells. BMB Rep. 2017;50(3):132–7. doi: 10.5483/bmbrep.2017.50.3.189 27931517 PMC5422025

[50] Hernández Borrero LJ, El-Deiry WS. Tumor suppressor p53: Biology, signaling pathways, and therapeutic targeting. Biochim Biophys Acta Rev Cancer. 2021;1876(1):188556. doi: 10.1016/j.bbcan.2021.188556 33932560 PMC8730328

[51] Xiao B-D, Zhao Y-J, Jia X-Y, Wu J, Wang Y-G, Huang F. Multifaceted p21 in carcinogenesis, stemness of tumor and tumor therapy. World J Stem Cells. 2020;12(6):481–7. doi: 10.4252/wjsc.v12.i6.481 32742565 PMC7360995

[52] Dong Y-X, Pang Z-G, Zhang J-C, Hu J-Q, Wang L-Y. Long non-coding RNA GClnc1 promotes progression of colorectal cancer by inhibiting p53 signaling pathway. Eur Rev Med Pharmacol Sci. 2019;23(13):5705–13. doi: 10.26355/eurrev_201907_18308 31298323

