# Supplementary figures and images for: The role of SLC2A1 in lung adenocarcinoma: From tumorigenesis to patient survival
Source: PLoS One. 2025 Aug 18;20(8):e0324043. doi: 10.1371/journal.pone.0324043 (PMC12360529; doi:10.1371/journal.pone.0324043)

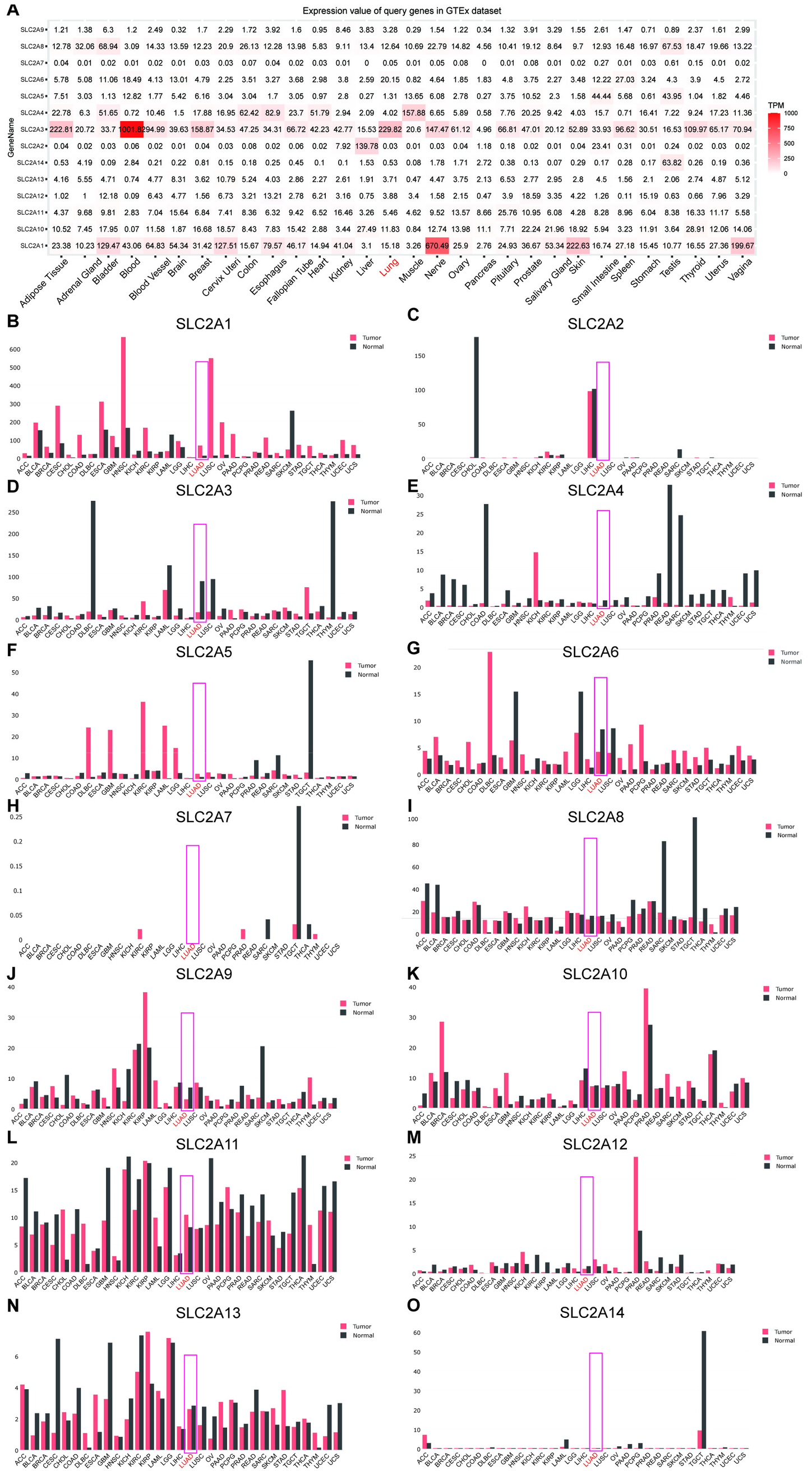

Supplement: S1 Fig — (A) Heat map: SLC2A expression in human normal tissues and organs in the GSCA database: (B–K) expression profile of SLC2A1–10 and (L–O) median expression levels of SLC2A11–14. (TIF) [file pone.0324043.s001.tif]

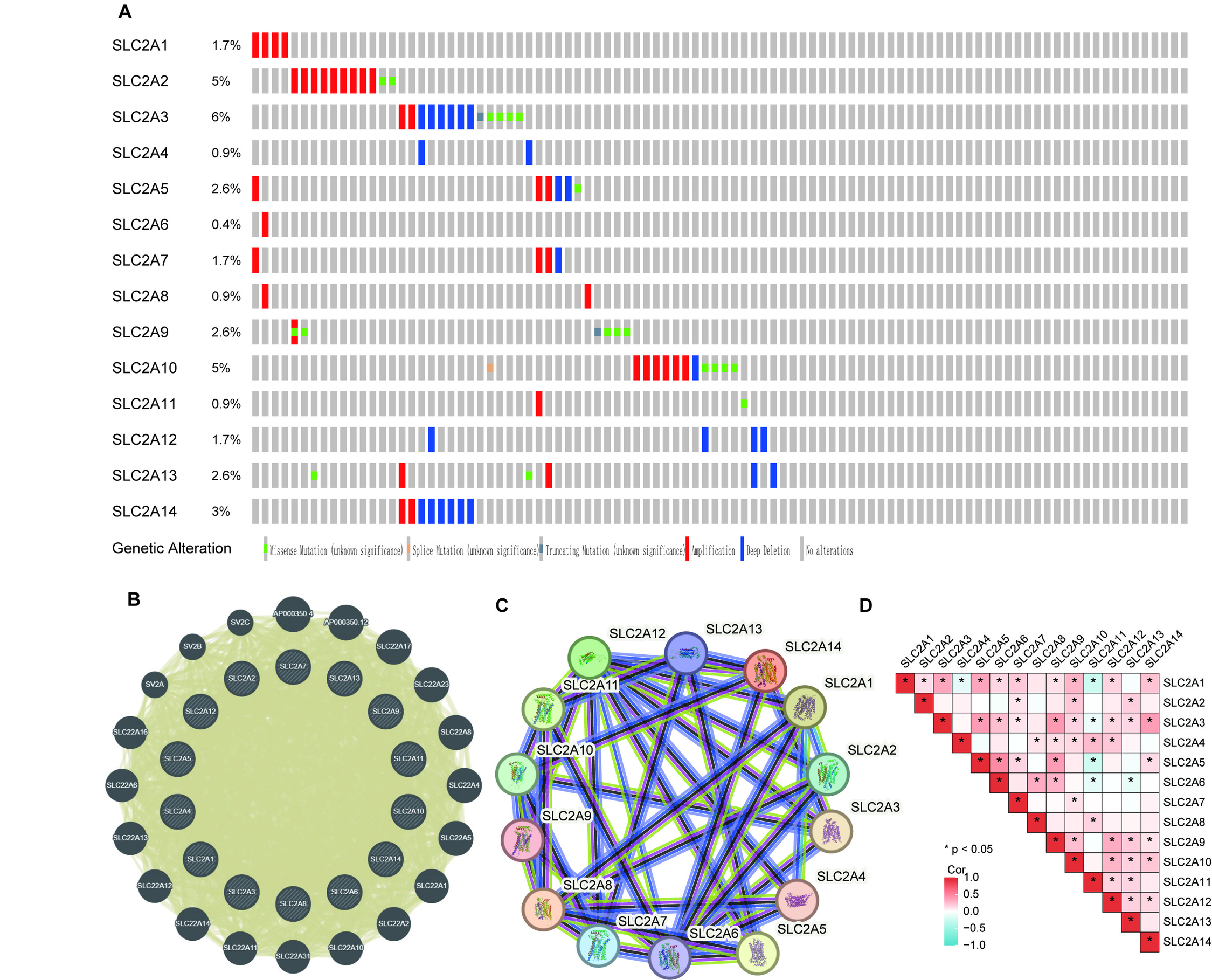

Supplement: S2 Fig — (A) Genetic variation of the SLC2A family in LUAD based on the cBioPortal database. (B–C) PPI network of SLC2A family genes by GeneMANIA and STRING databases. (D) Correlation analysis among SLC2A family proteins in LUAD based on the Xiantao Academic database. LUAD: Lung adenocarcinoma. PPI: Protein-protein interaction. (TIF) [file pone.0324043.s002.tif]

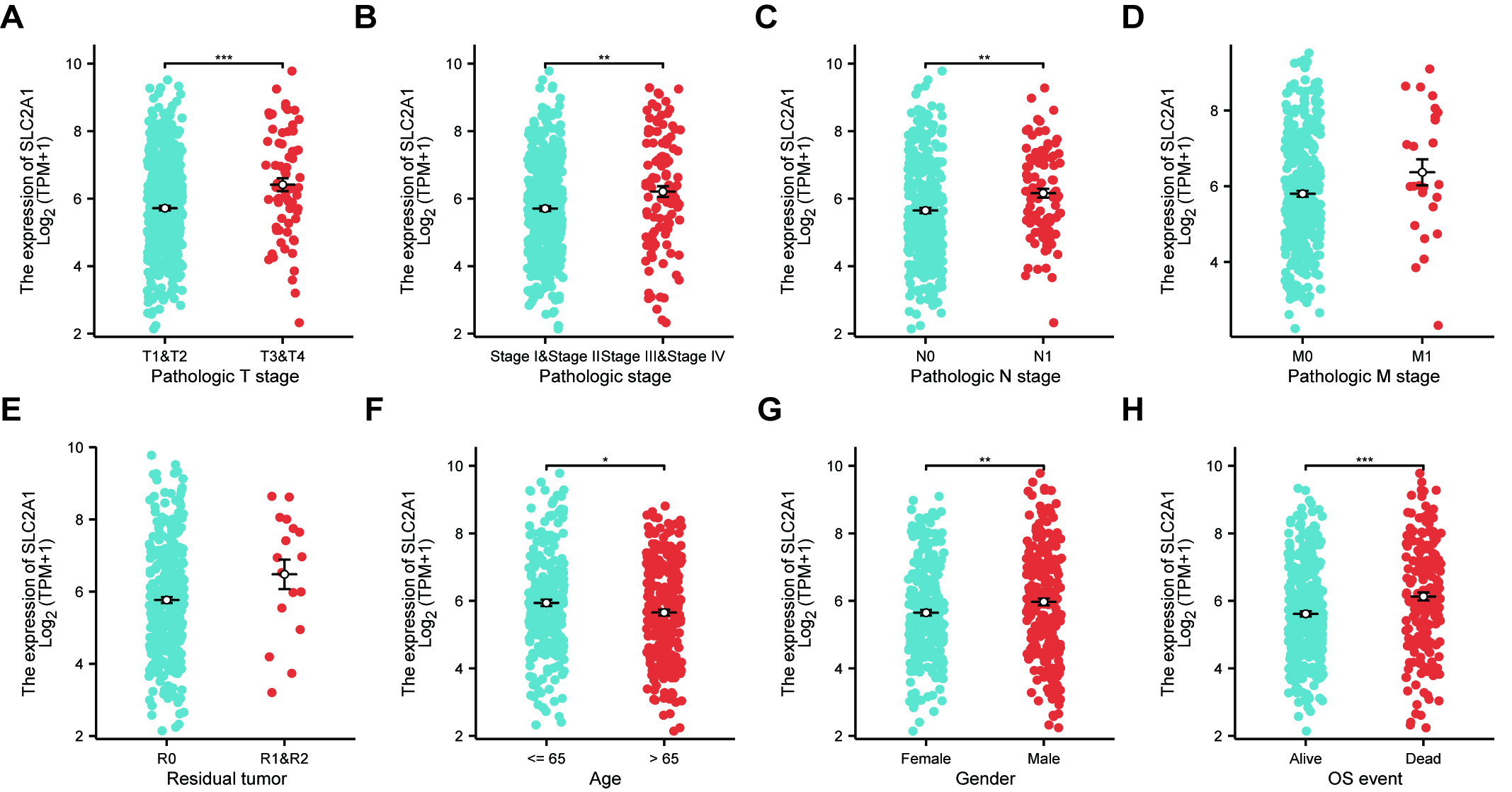

Supplement: S3 Fig — (A) Pathological T stage. (B) Pathologic stage. (C) Pathologic N stage. (D) Pathologic M stage. (E) Residual tumor. (F) Age. (G) Gender. (H) OS events. (TIF) [file pone.0324043.s003.tif]

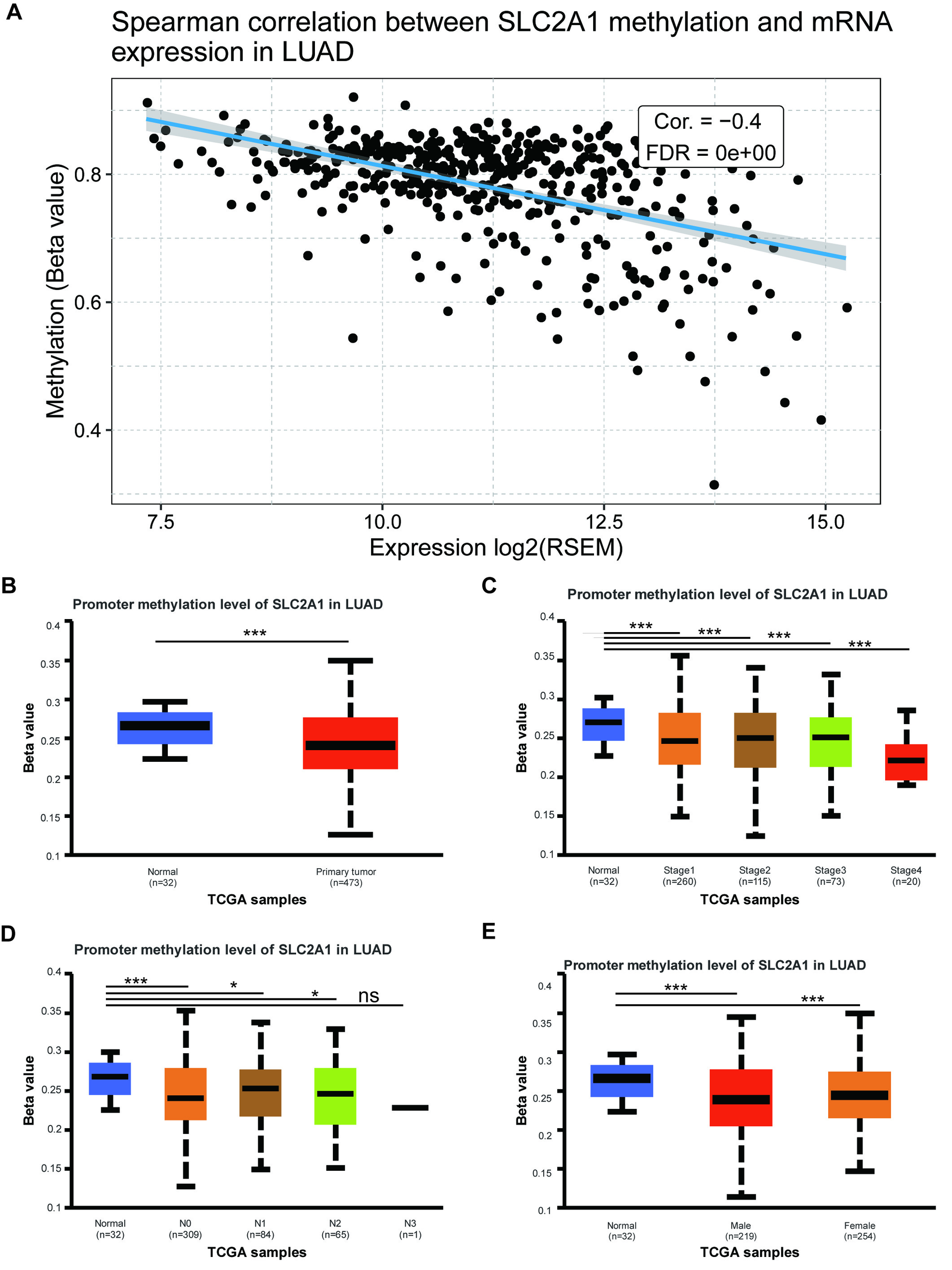

Supplement: S4 Fig — (A) Correlation between SLC2A1 methylation in LUAD and SLC2A1 mRNA expression. (B) Methylation levels of the SLC2A1 gene in LUAD tissues. (C) Correlation between the stage of LUAD patients and methylation status of the SLC2A1 gene. (D) Correlation between N stage and SLC2A1 methylation in LUAD patients. (E) Relation between gender-specific methylation patterns and expression levels of SLC2A1 in LUAD patients. (TIF) [file pone.0324043.s004.tif]

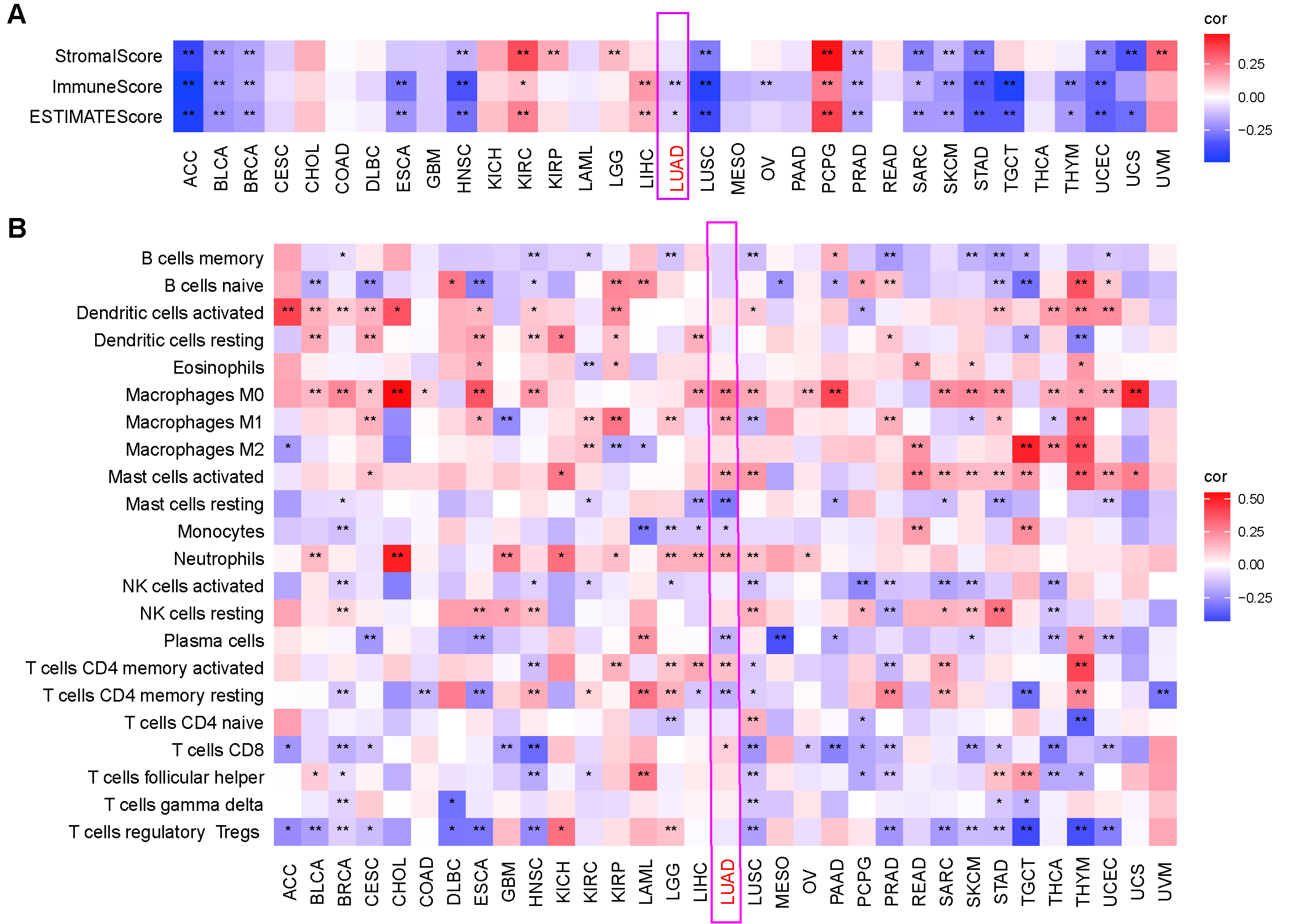

Supplement: S5 Fig — (A) Correlation between SLC2A1 expression and tumor microenvironment and (B) immune cell infiltration in various tumors. *P < 0.05; **P < 0.01; ***P < 0.001. (TIF) [file pone.0324043.s005.tif]

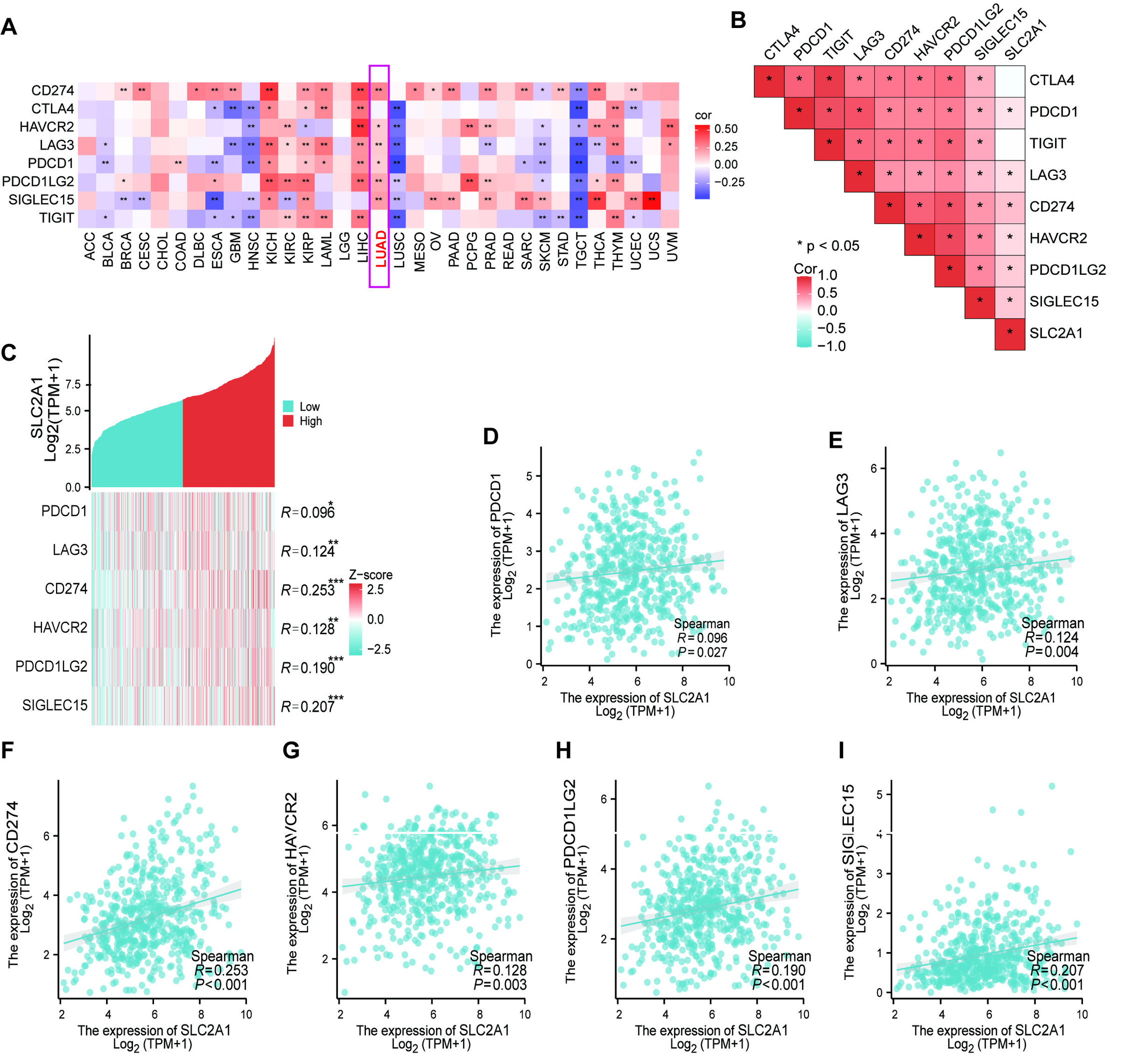

Supplement: S6 Fig — (A) Correlation between SLC2A1 expression and immune checkpoint in 33 tumors. (B) Heat maps: Correlation between SLC2A1 expression and immune checkpoint. (C) Correlation of SLC2A1 expression with PDCD1, LAG3, CD274, HAVCR2, PDCD1LG2 and SIGLEC15; Scatter plots: Positively correlation of SLC2A1 expression with PDCD1 (D), LAG3 (E), CD274 (F), HAVCR2 (G), PDCD1LG2 (H) and SIGLEC15 (I). (TIF) [file pone.0324043.s006.tif]

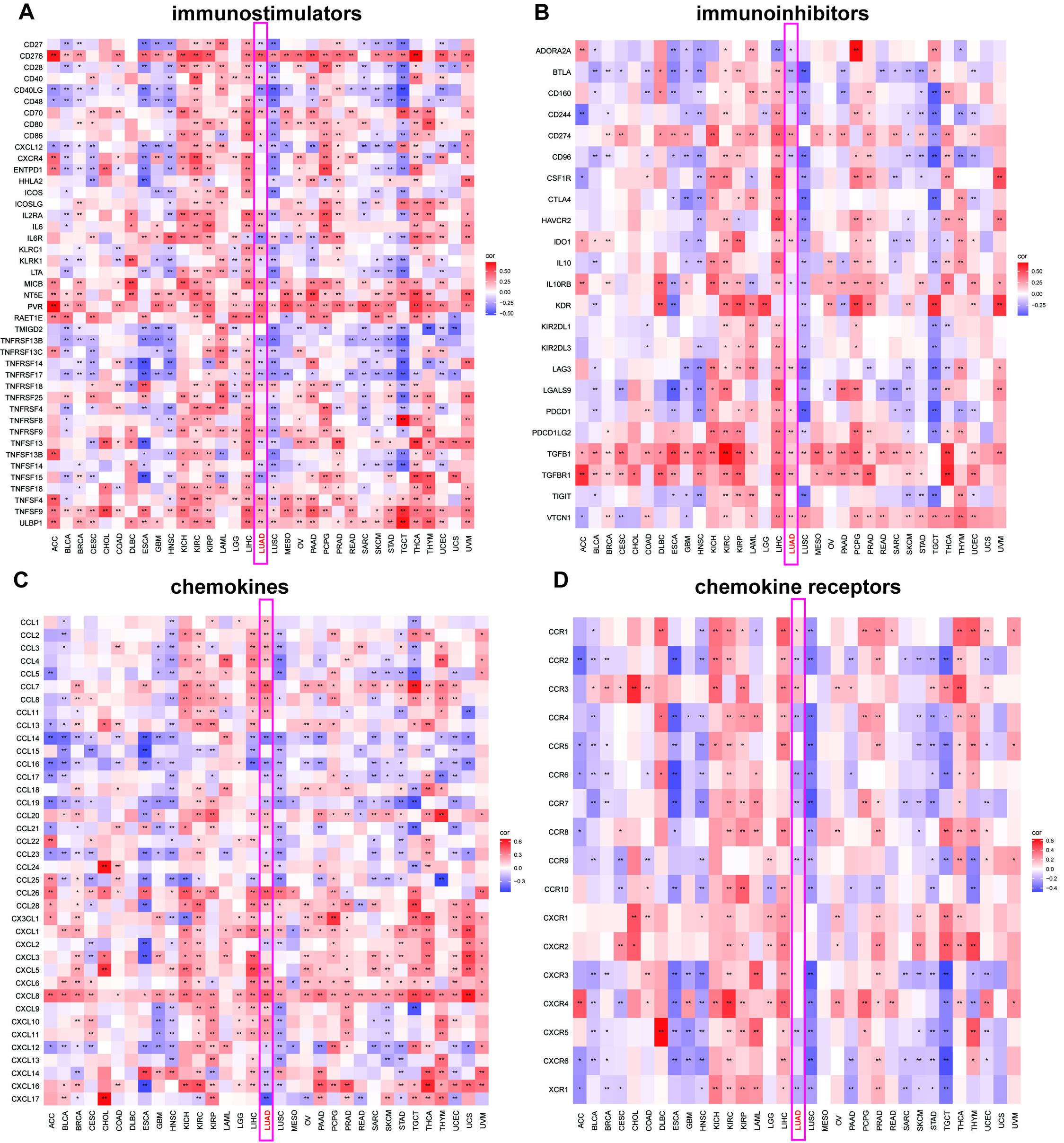

Supplement: S7 Fig — (A) Heat map: Correlation analysis between SLC2A1 expression and immunosuppressive agents and (B) immunostimulants. (C) Heat map: Correlation analysis between SLC2A1 expression and chemokines and (D) chemokine receptors. (TIF) [file pone.0324043.s007.tif]
