# Supplementary material for: The role of SLC2A1 in lung adenocarcinoma: From tumorigenesis to patient survival
Source: PLoS One. 2025 Aug 18;20(8):e0324043. doi: 10.1371/journal.pone.0324043 (PMC12360529; doi:10.1371/journal.pone.0324043)
Supplement: S1 Data — (PDF) [file pone.0324043.s008.pdf]

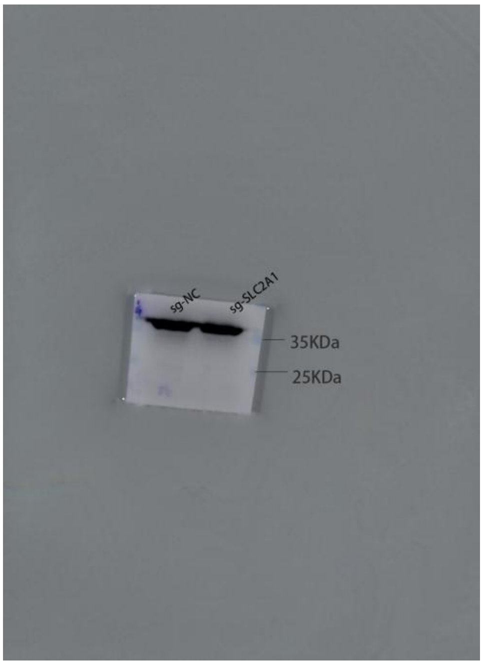

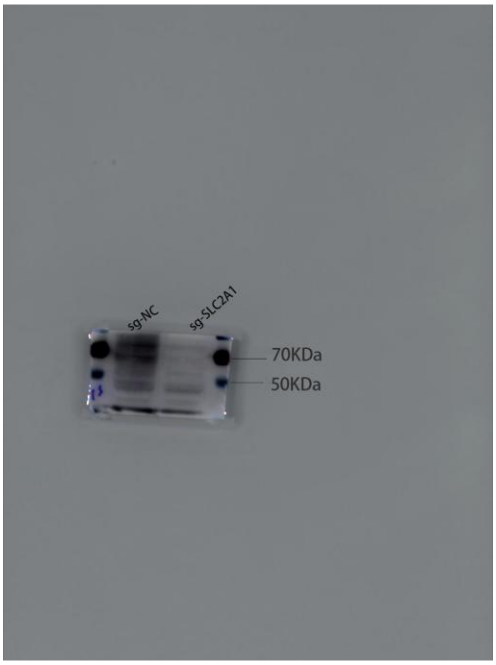

GAPDH SLC2A1

These two graphs are used to generate Fig 6I

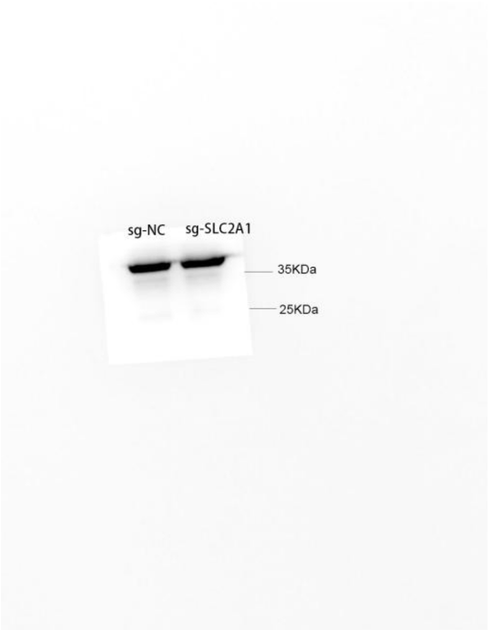

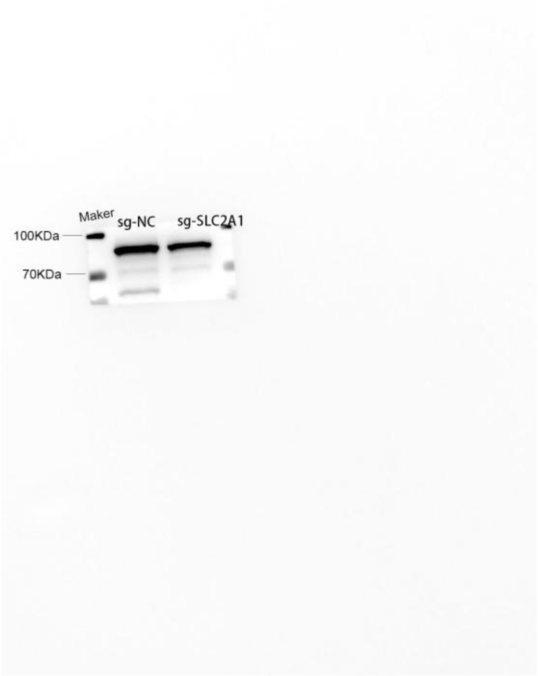

GAPDH E-cadherin

These two graphs are used to generate Fig 8D.

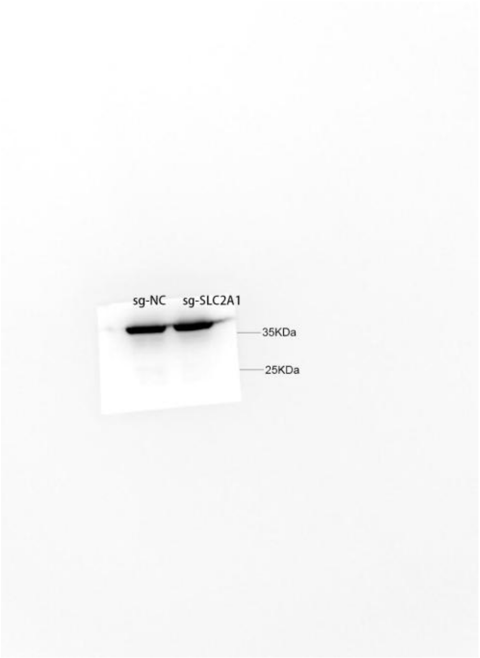

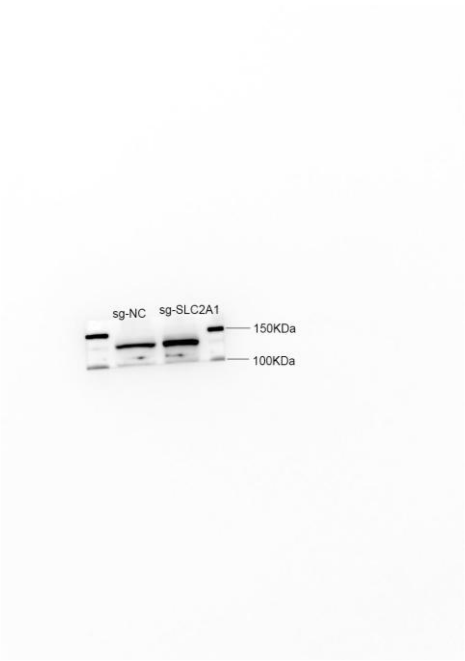

GAPDH

N-cadherin

These two graphs are used to generate Fig 8E.

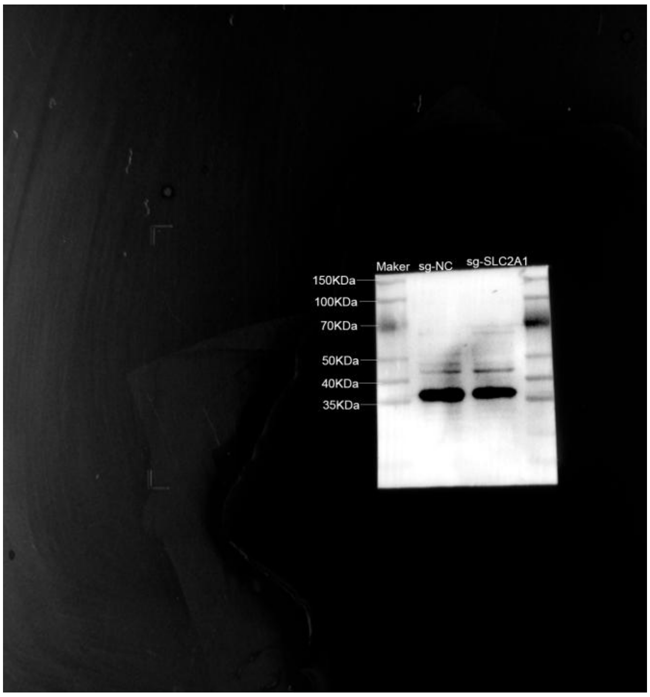

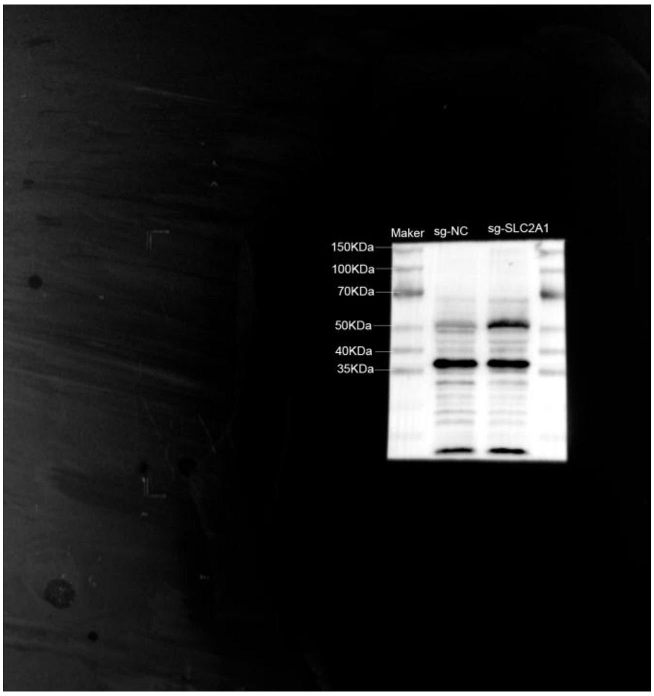

GAPDH

P53

These two graphs are used to generate the internal reference gene and P53 expression image in Fig 10B.

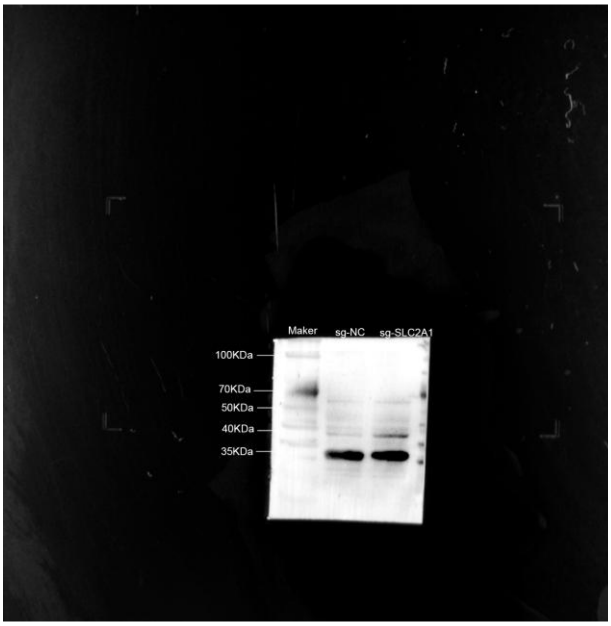

GAPDH

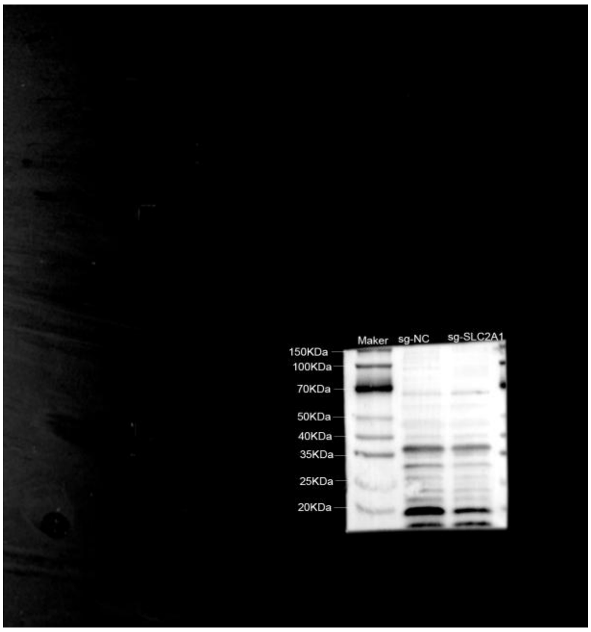

P21

These two graphs are used to generate the internal reference gene and P21 expression image in Fig 10B.

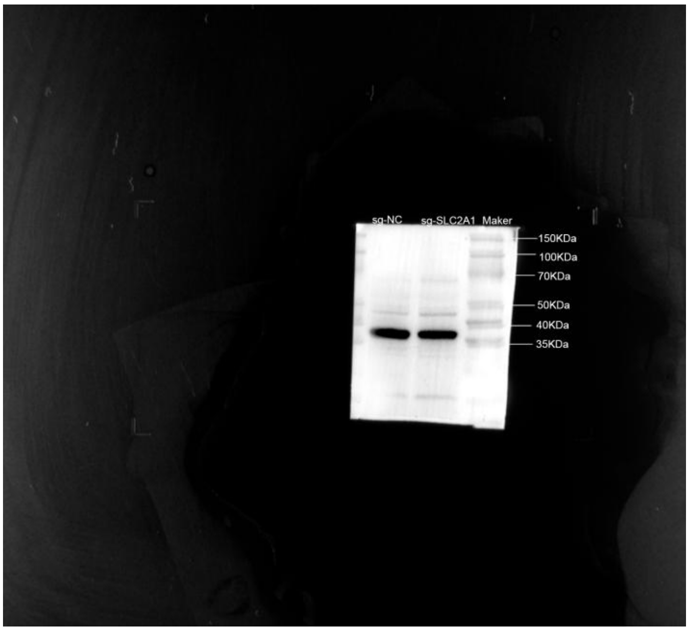

GAPDH

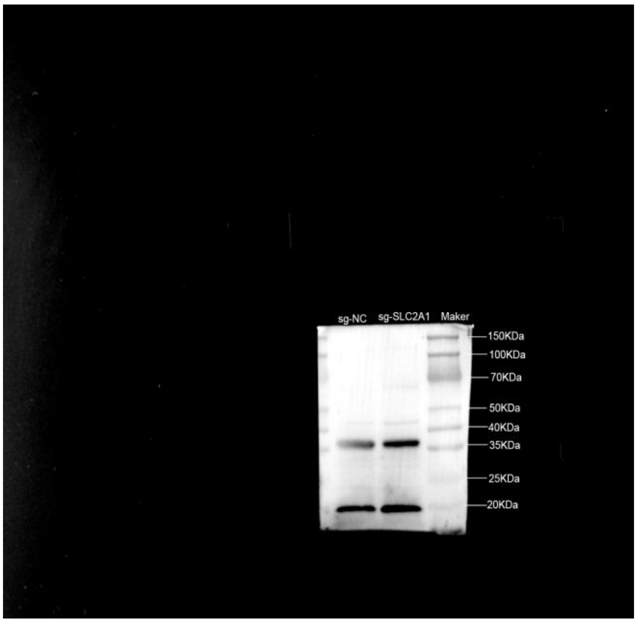

BAX

These two graphs are used to generate the internal reference gene and BAX expression image in Figure 10B.

All of the above Western Blot images were imaged by chemiluminescence, and labeled every image in the file to annotate the loading order.
